# Supplementary material for: Elucidating molecularly stratified single agent, and combination, therapeutic strategies targeting MCL1 for lethal prostate cancer
Source: Nat Commun. 2025 Oct 8;16:8806. doi: 10.1038/s41467-025-64042-5 (PMC12508096; doi:10.1038/s41467-025-64042-5)
Supplement: Supplementary file 1 — Supplementary Information [file 41467_2025_64042_MOESM1_ESM.pdf]

1) Supplementary Figures Page 1

2) Supplementary Tables Page 33

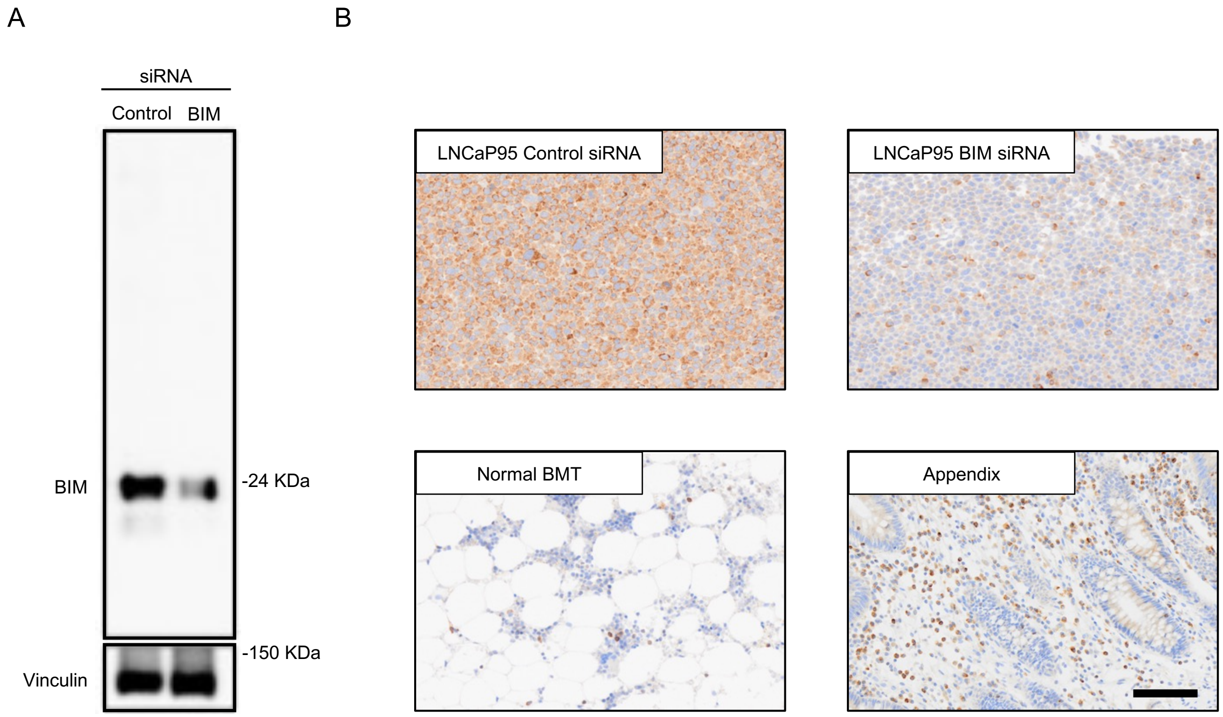

Supplementary Figure 1. BIM antibody validation and immunohistochemistry optimisation. (A-B) Representative western blot **(A)** and immunohistochemistry **(B)** of BIM detection using the #2933 (Cell Signaling Technology) antibody in LNCaP95 cells treated with control or BIM siRNA (50 nM; 72h). Bone marrow trephine (BMT) and appendix are used as controls. The scale bar indicates a length of 100 µm.

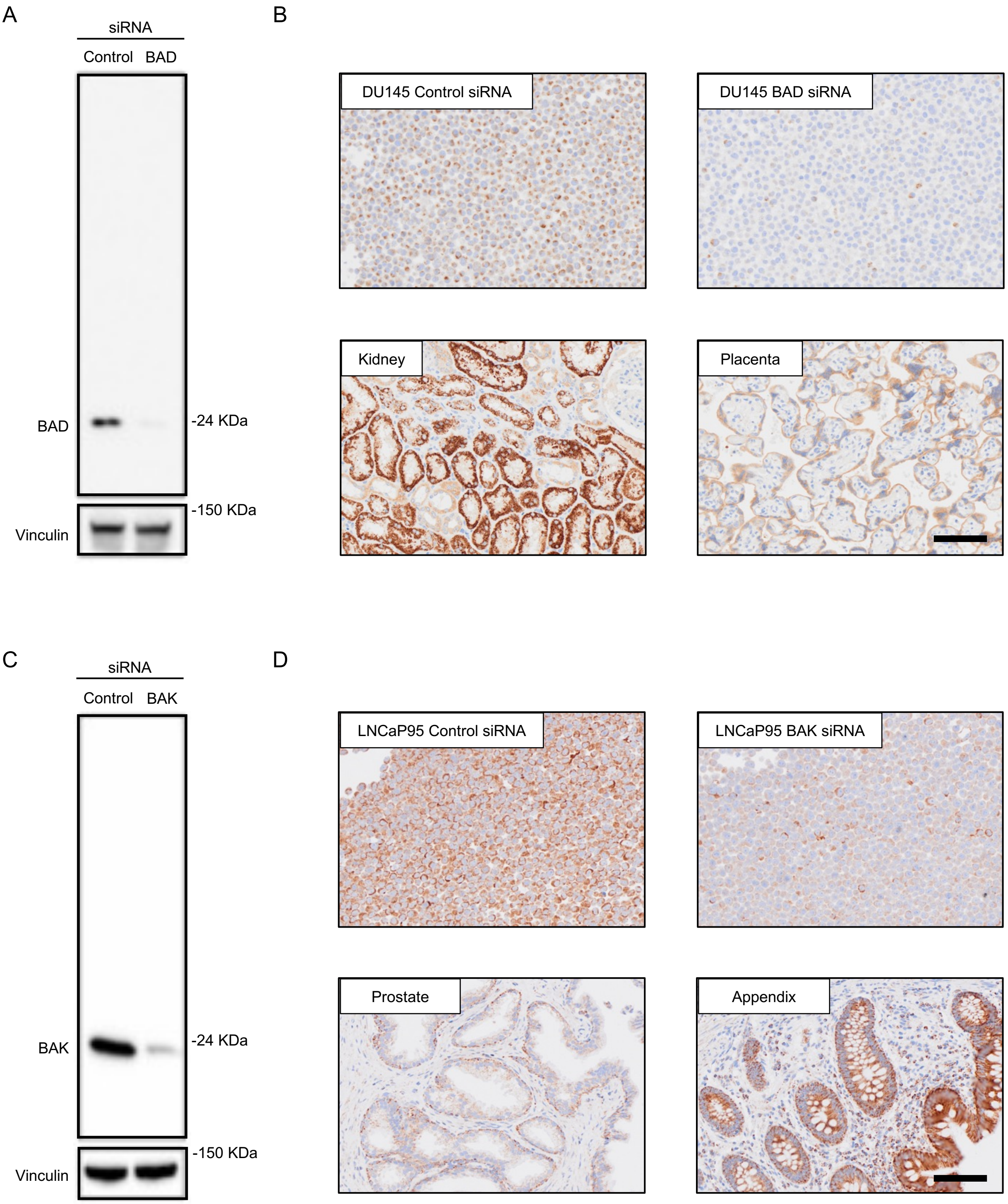

Supplementary Figure 2. BAD and BAK antibody validation and immunohistochemistry optimisation. (A-B) Representative western blot **(A)** and immunohistochemistry **(B)** of BAD detection using the ab32445 (Abcam) antibody in DU145 cells treated with control or BAD siRNA (50 nM; 72h). Kidney and placenta tissues are used as controls. The scale bar indicates a length of 100 µm. **(C-D)** Representative western blot **(C)** and immunohistochemistry **(D)** of BIM detection using the #12105 (Cell Signaling Technology) antibody in LNCaP95 cells treated with control or BAK siRNA (50 nM; 72h). Prostate and appendix tissues are used as controls. The scale bar indicates a length of 100 µm.

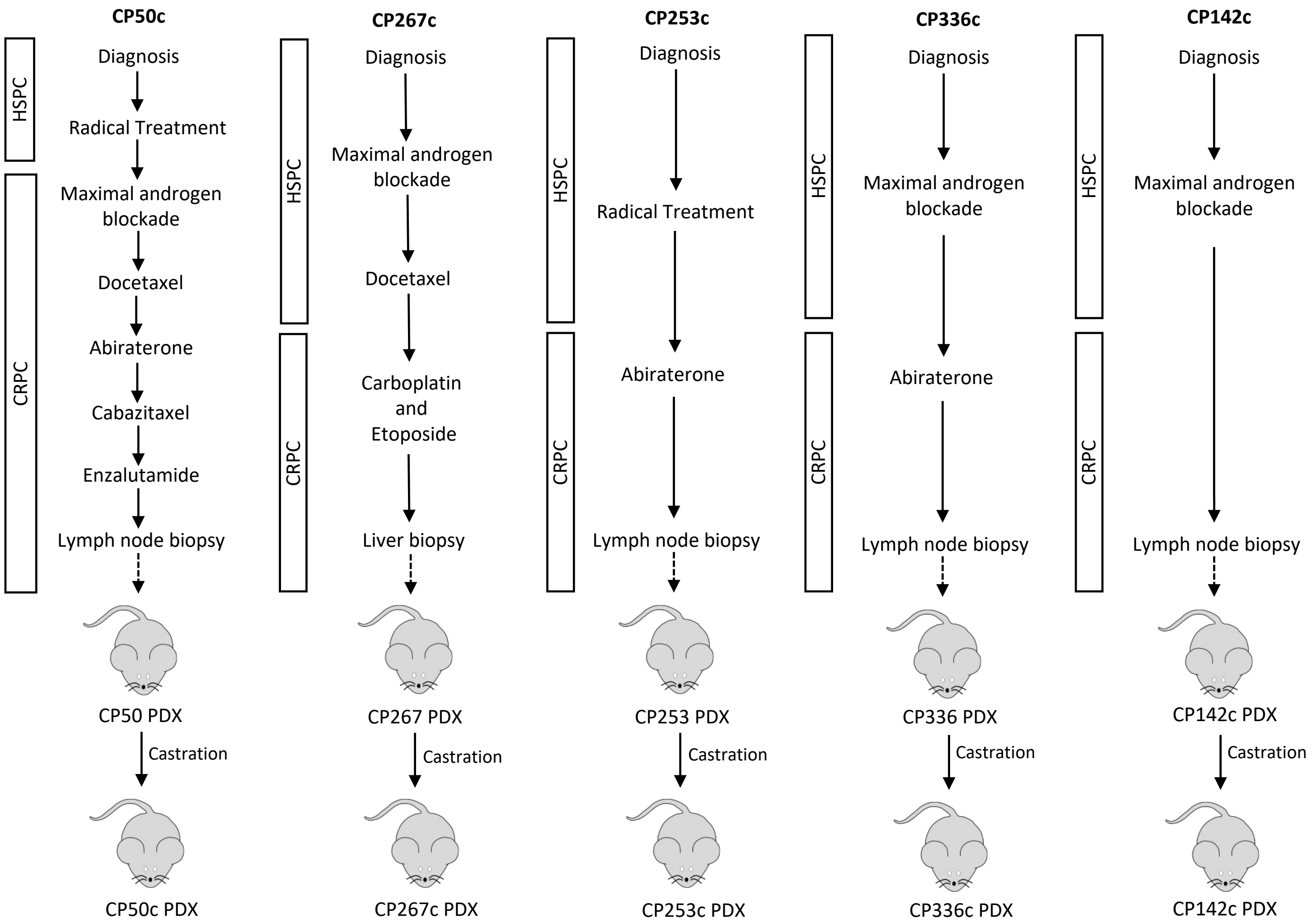

Supplementary Figure 3. Clinical history for patients whose biopsies were used to generate patient-derived xenografts. Patient derived xenografts (PDXs) were stablished from human CRPC biopsies. HSPC: Hormone sensitive prostate cancer. CRPC: Castration resistant prostate cancer. PDX: Patient-derived xenograft.

Supplementary Figure 4

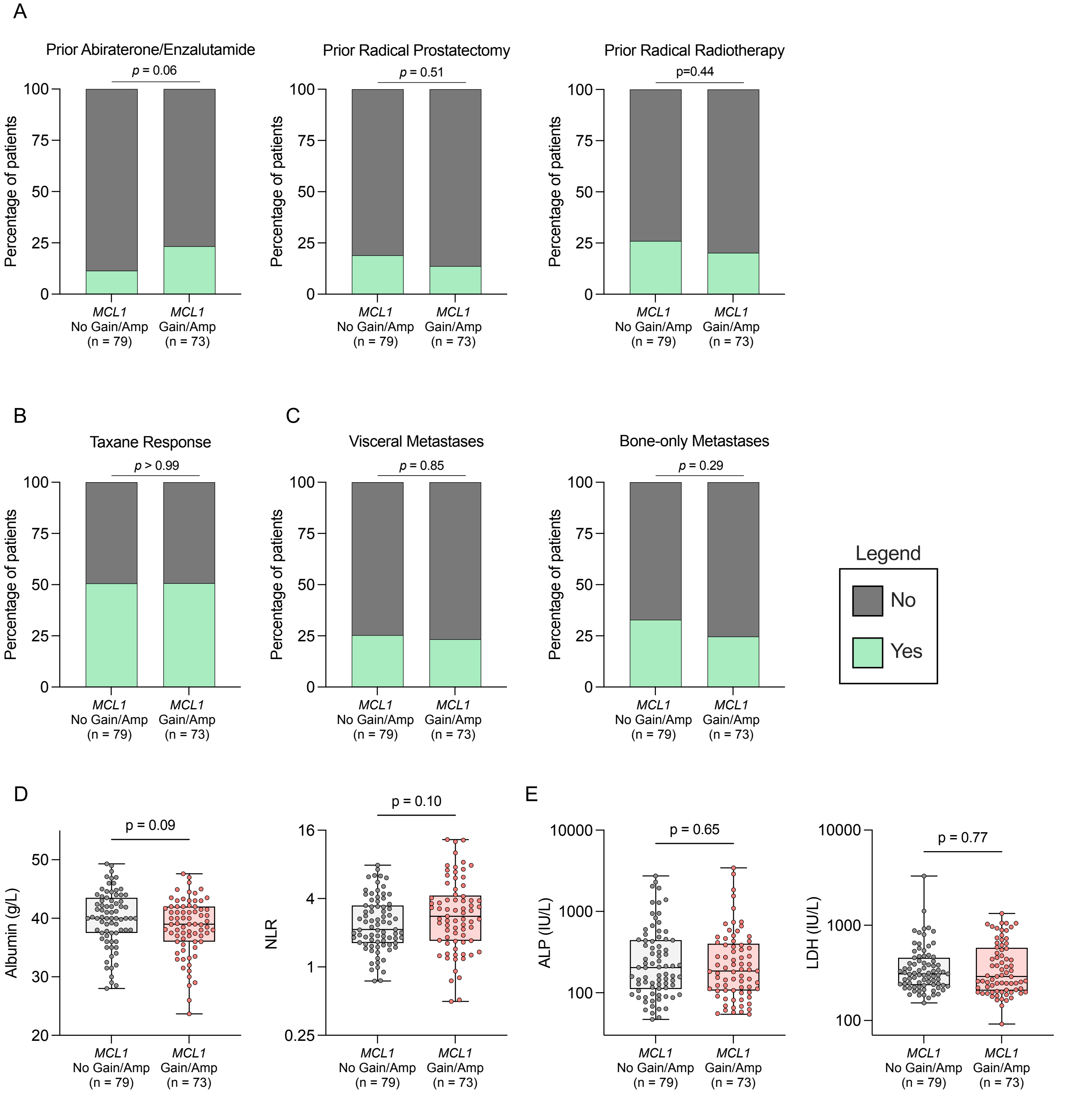

Supplementary Figure 4. Associations between *MCL1* copy number and clinical parameters in the FIRSTANA/PROSELICA cohort. (A) The percentage of patients, with and without *MCL1* copy number gain/amplification, previously treated with abiraterone and/or enzalutamide (left hand-side panel), radical radiotherapy (central panel) and radical prostatectomy (right hand-side panel). The p value was calculated using the Fisher's exact test. **(B-C)** The percentage of patients, with and without *MCL1* copy number gain/amplification, who had a response to taxane chemotherapy **(B)**, and had visceral (left hand-side panel) or bone-only metastases (right hand-side panel) **(C)**. The p value was calculated using the Fisher's exact test. **(D-E)** Comparison of albumin levels (g/L; left hand-side panel), neutrophil-to-lymphocyte ratio (NLR; right hand-side panel) **(D)**, Serum alkaline phosphatase (ALP; IU/L; left hand-side panel), and Lactate dehydrogenase (LDH; IU/L; right hand-side panel) **(E)** between patients with and without *MCL1* copy number gain/amplification. The median and IQRs are shown. The p value was calculated using the Mann-Whitney U test.

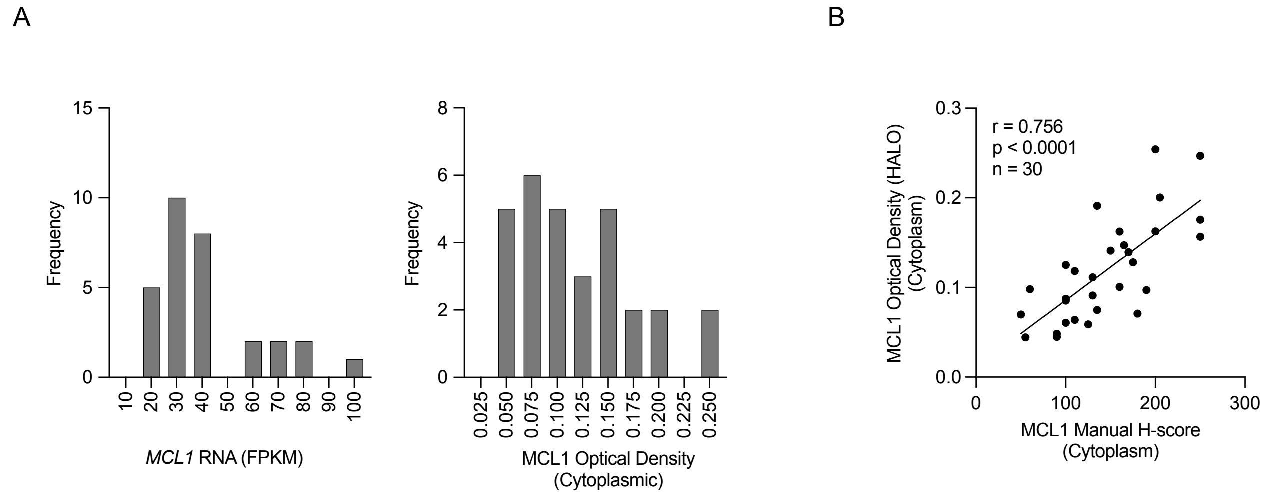

Supplementary Figure 5. MCL1 RNA and protein expression in CRPC Cohorts. (A) Histogram showing distribution of *MCL1* RNA expression (left hand side panel). Histogram showing distribution of MCL1 protein expression measured as optical density (OD; right hand-side panel). **(B)** Spearman correlation between MCL1 optical density and MCL1 manual H-Score values in 30 samples from the RMH CRPC cohort.

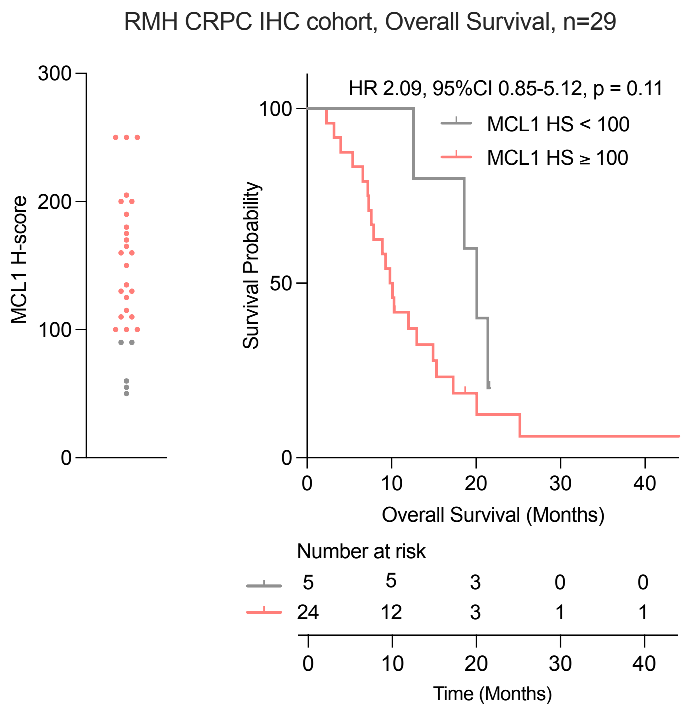

Supplementary Figure 6: Association of MCL1 protein expression with overall survival in CRPC patients. (Left) Dot plot representing MCL1 protein levels (H-Score) measured in a cohort of 29 CRPC patient samples. Each dot represents an individual patient, with red dots indicating tumours with MCL1 H-Score ≥ 100 and grey dots indicating tumours with H-Score < 100. (Right) Kaplan-Meier survival curves showing overall survival from biopsy, stratified by MCL1 protein expression levels. Patients with MCL1 H-Score ≥ 100 are represented by the red line, and those with H-Score < 100 by the grey line. Hazard ratio (HR) with 95% confidence intervals and p-value for log-rank test are shown. HS: H-Score.

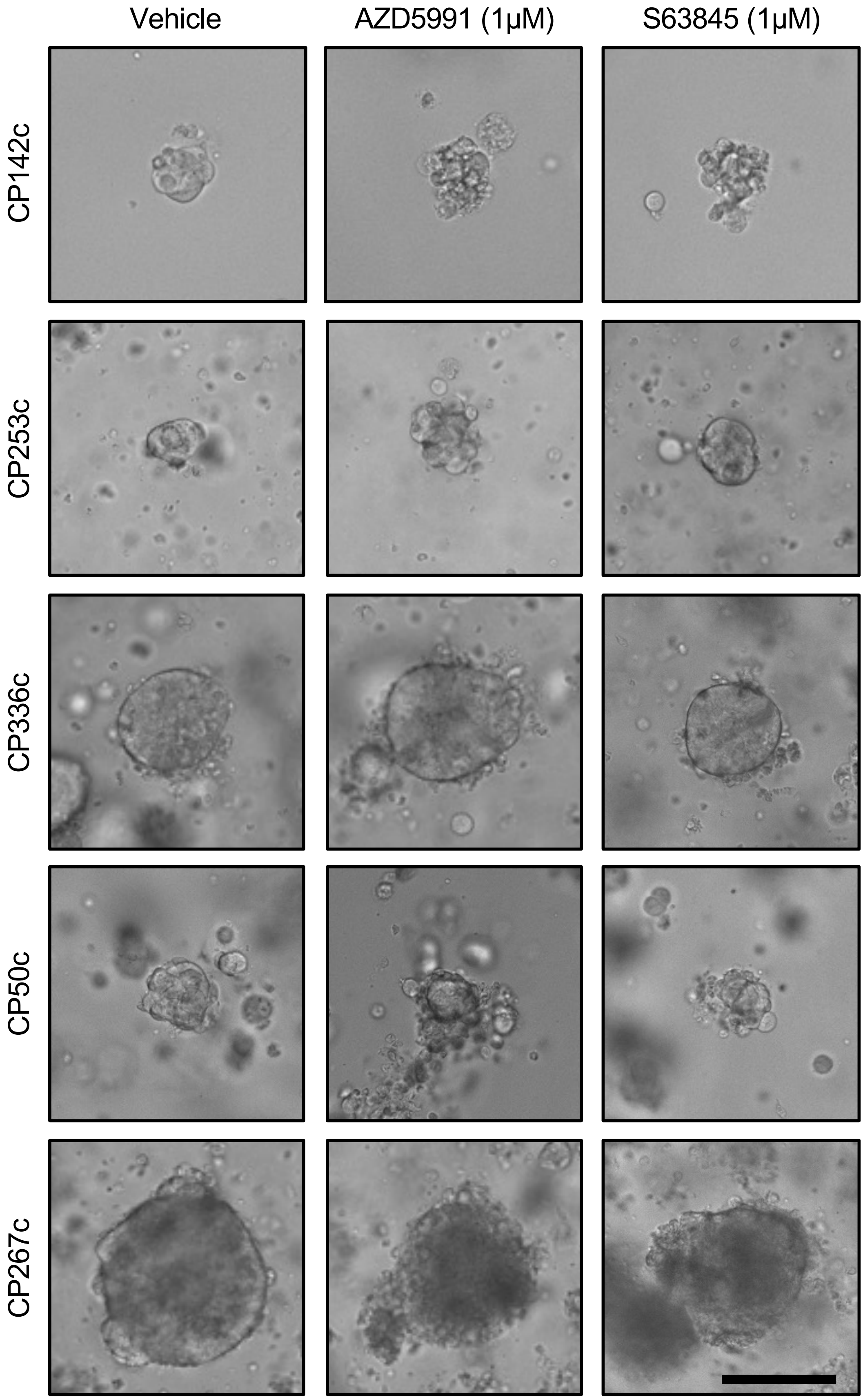

Supplementary Figure 7. Representative microscopy images of CP142c, CP253c, CP336c, CP50c and CP267c CRPC PDX-Os treated with AZD5991 or S63845. Drugs were used at 1μM. Images were taken on day 4 after treatment. The scale bar indicates a length of 200 μm.

Supplementary Figure 8

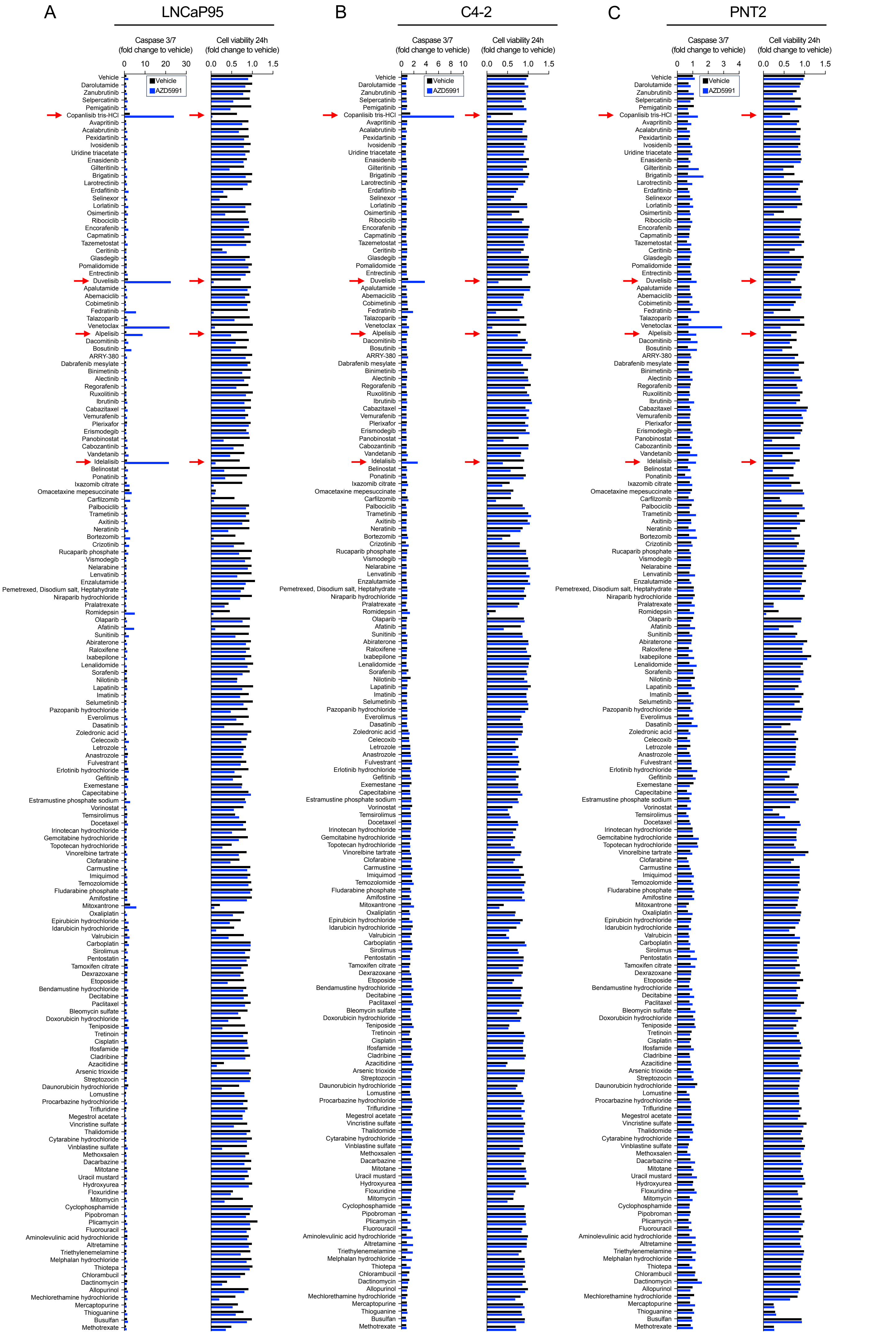

Supplementary Figure 8. Effect of 166 FDA-approved drugs in absence and presence of AZD5991 on caspase 3/7 activity and cell viability. Caspase 3/7 activity was measured at 6 hours using Caspase-Glo 3/7 2D, and cell viability at 24 hours using CellTiter-Glo 2D. FDA-approved drugs were tested at 5 µM, and AZD5991 at 1 µM. Black bars represent FDA-approved single-agent effects, and blue bars represent effects in combination with AZD5991. The screen was conducted as a single biological experiment (n=1) without technical replicates.

Supplementary Figure 9

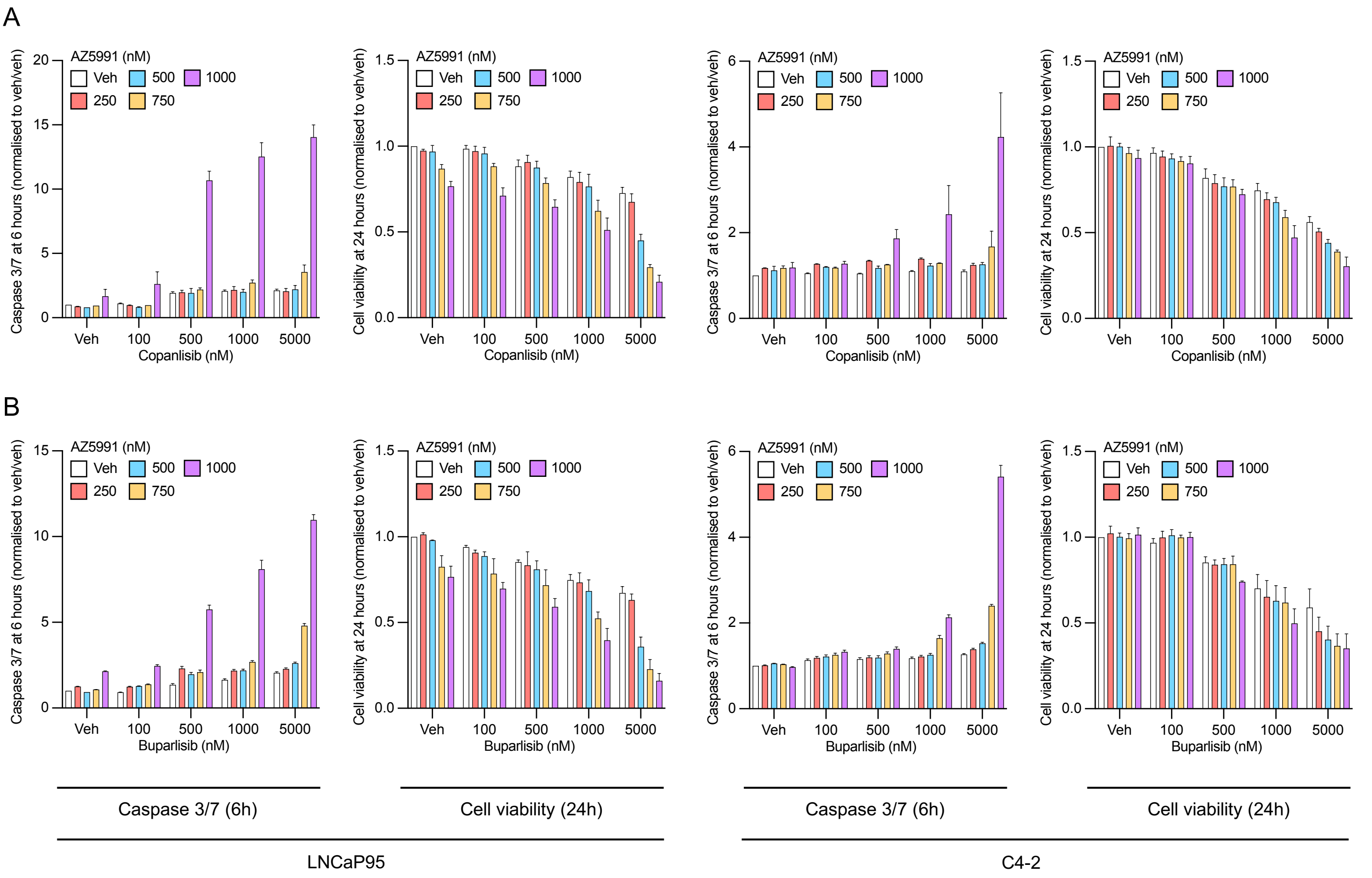

Supplementary Figure 9. Co-inhibition of PI3K and MCL1 drives apoptotic cell death in a dose-dependent manner in CRPC cells. Caspase 3/7 levels (at 6 hours; Caspase-Glo 3/7 assay) and cell viability (at 24 hours; CellTiter-Glo assay) are shown in response to varying concentrations of AZD5991 in absence or presence of varying concentrations of copanlisib (A) and buparlisib (B) in LNCaP95 and C4-2 cells. All the experiments were performed in three biological triplicates and the standard error of the mean is shown.

Supplementary Figure 10

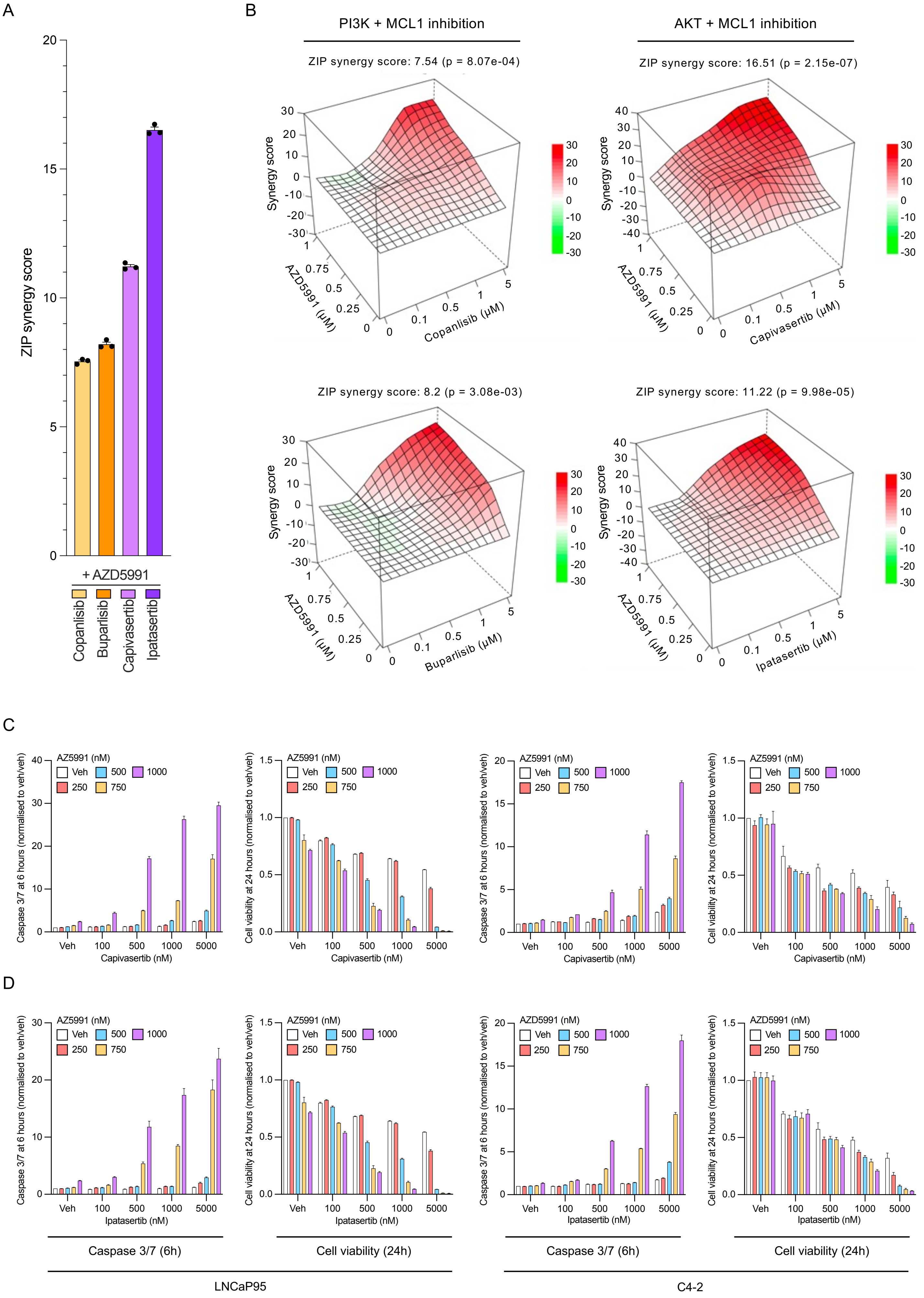

Supplementary Figure 10. AKT and MCL1 co-inhibition exhibits stronger synergistic effects on apoptosis and cell death induction compared to PI3K and MCL1 co-inhibition in CRPC cells. (A) Comparison of Zero Interaction Potency (ZIP) synergy scores for AZD5991 combined with PI3K inhibitors (copanlisib, buparlisib) versus AZD5991 combined with AKT inhibitors (capivasertib, ipatasertib), in C4-2. Data represent three biological triplicates with three technical replicates; error bars show Standard Error of the Mean. One-way ANOVA with post-hoc Tukey test was performed. **(B)** ZIP synergy score surface plots of AZD5991 combined with PI3K inhibitors (copanlisib and buparlisib; left panels) and AKT inhibitors (capivasertib and ipatasertib; right panels) in C4-2. **(C-D)** Caspase 3/7 levels (at 6 hours; Caspase-Glo 3/7 assay) and cell viability (at 24 hours; CellTiter-Glo assay) are shown in response to varying concentrations of AZD5991 in absence or presence of varying concentrations of capivasertib **(C)**, ipatasertib **(D)** in LNCaP95 and C4-2 cells. All the experiments were performed in three biological triplicates and the standard error of the mean is shown.

Supplementary Figure 11

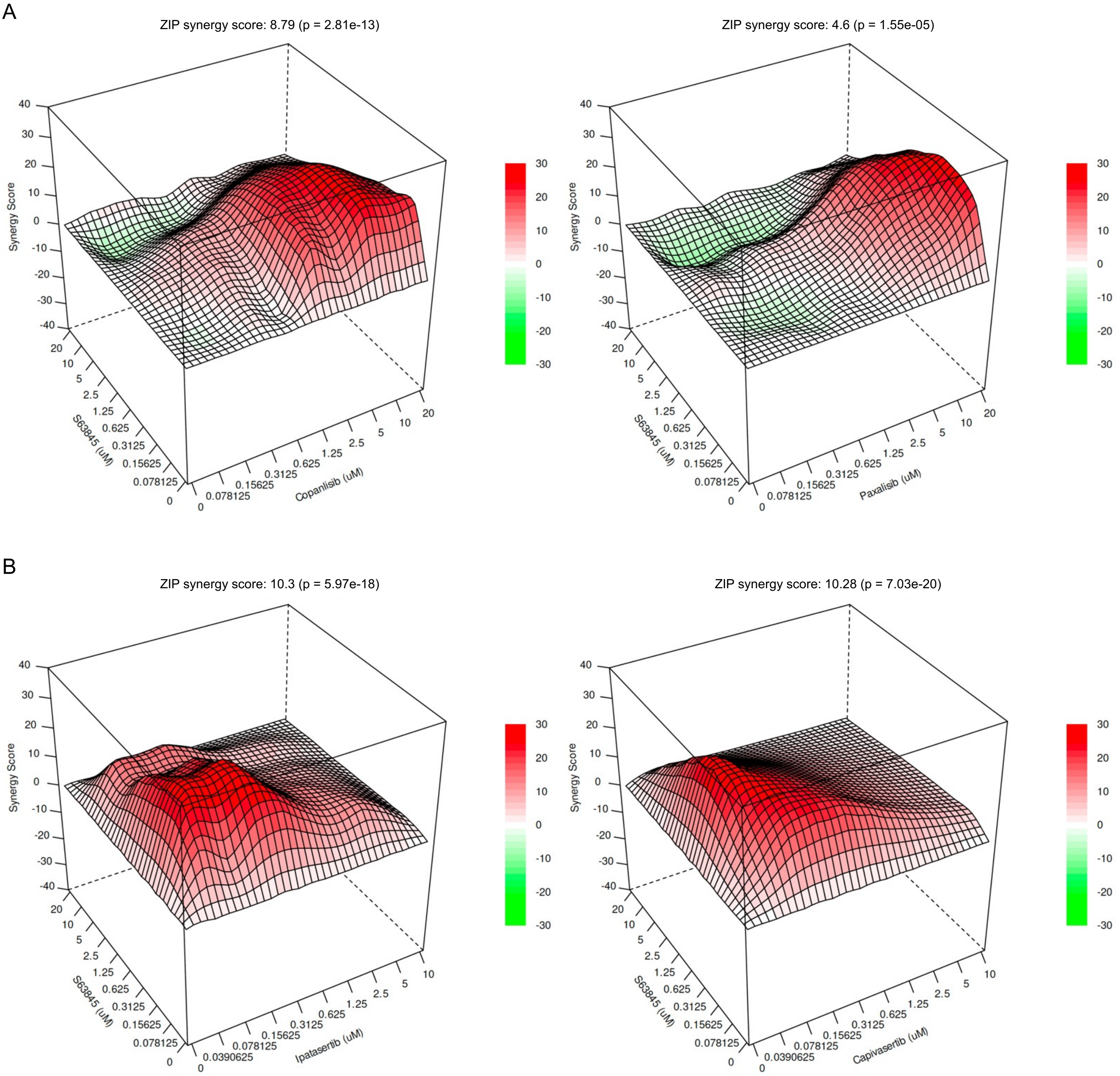

Supplementary Figure 11. PI3K and AKT inhibitors synergise with MCL1 inhibition to induce apoptotic cell death in CRPC cell lines. (A-B) Zero Interaction Potency (ZIP) synergy score surface plots of S63845 combined with PI3K inhibitors (copanlisib and paxalisib) **(A)** and AKT inhibitors (capivasertib and ipatasertib) **(B)** in LNCaP95 cells. Data from a high-throughput combination matrix drug screening (861 different combinations), carried out independently at the NCI.

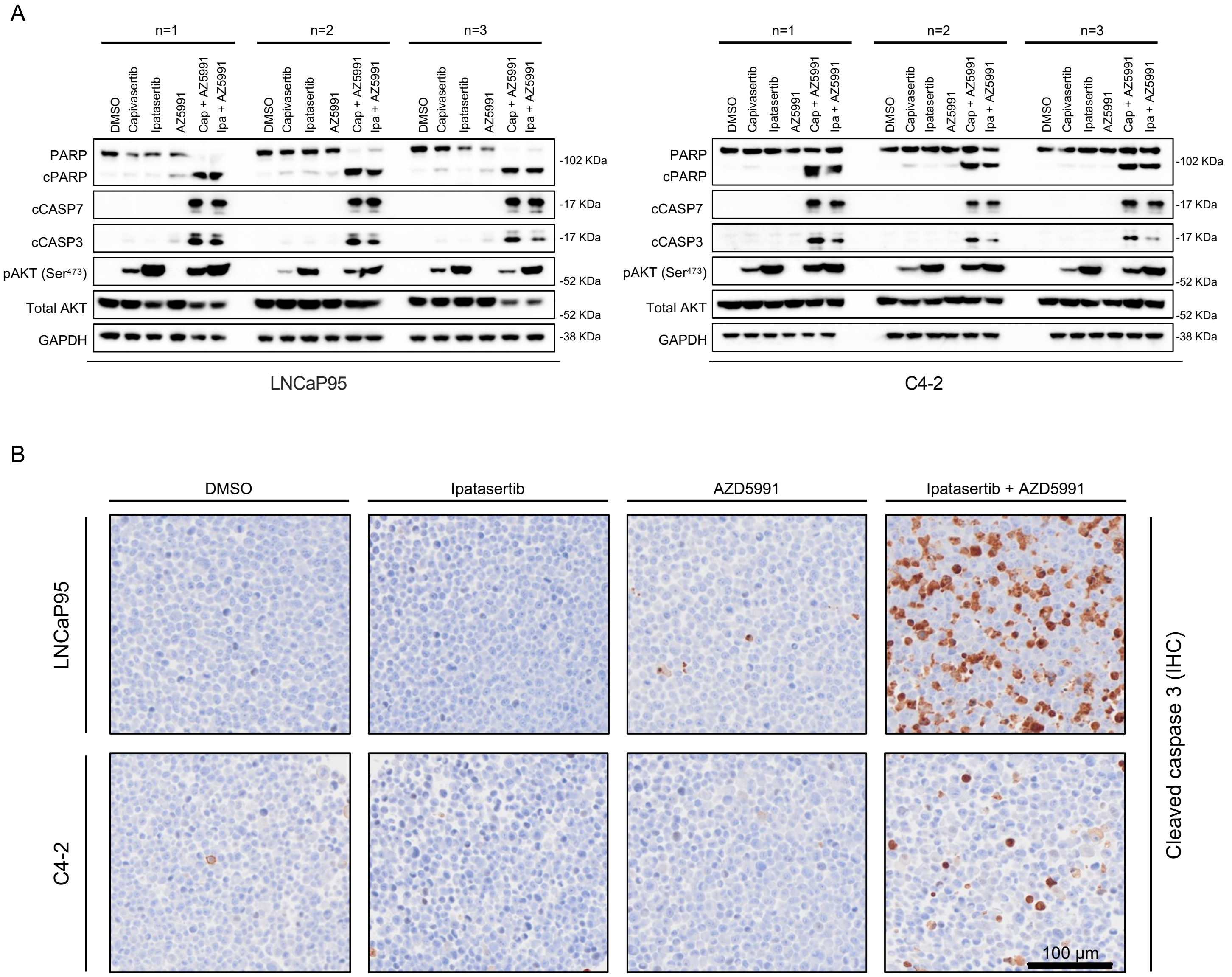

Supplementary Figure 12. PI3K and AKT inhibitors synergise with MCL1 inhibition to induce apoptotic cell death in CRPC cell lines. (A) Comparison of protein expression of cleaved PARP, cleaved caspase 3, total and p-AKT^{Ser473} (at 6 hours) between vehicle (DMSO), capivasertib (1μM), ipatasertib (1μM), and combined treatments in LNCaP95 (left hand-side panels) and C4-2 (right hand-side panels). The experiment was performed in three biological triplicates in technical singlets. **(B)** Representative micrographs showing cleaved caspase 3 levels by immunohistochemistry in response to vehicle (DMSO), ipatasertib, AZD5991 and combined treatment in LNCaP95 (top panel) and C4-2 (bottom panel). Drugs were used at 1μM. Cells were harvested and processed after 6 hours of treatment. The experiment was performed as a single biological replicate. The scale bar indicates a length of 100 μm.

Supplementary Figure 13

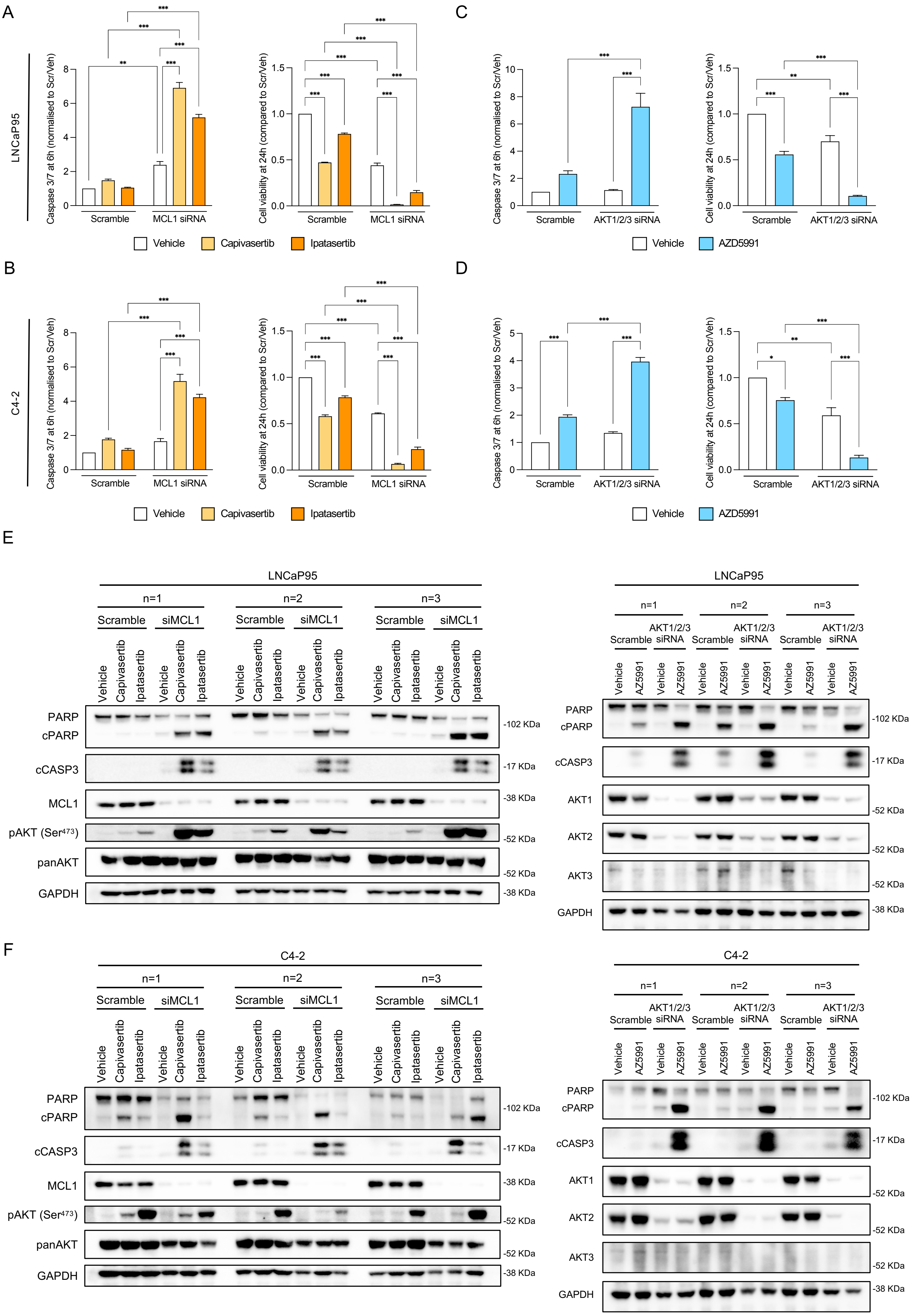

Supplementary Figure 13. AKT and MCL1 inhibition synergise with MCL1 and AKT silencing respectively to induce cell death in CRPC cell lines. (A-B) Caspase 3/7 (at 6 hours) levels and cell viability (at 24 hours) in response to capivasertib (1 µM) and ipatasertib (1 µM) upon transfection with MCL1 siRNA (25nM) or control siRNA (scramble; 25 nM) for 72 hours in LNCaP95 **(A)** and C4-2 **(B)**. One-way ANOVA with post-hoc Tukey was performed. **(C-D)** Caspase 3/7 (at 6 hours) levels and cell viability (at 24 hours) in response to vehicle and AZD5991 (1 µM) upon transfection with a cocktail of AKT1/2/3 siRNAs (25nM each) or scramble siRNA (75 nM) for 72 hours in LNCaP95 **(C)** and C4-2 **(D).** Caspase 3/7 activity and cell viability were determined using the Caspase-Glo 3/7 2D assay and the CellTiter-Glo 2D assay, respectively. One-way ANOVA with post-hoc Tukey was performed. **(E-F)** Protein levels of cleaved PARP, cleaved caspase 3, MCL1, total and p-AKT^{Ser473} (after 6 hours of treatment) in response to vehicle, capivasertib+AZD5991 and ipatasertib+AZD5991, upon transfection with MCL1 siRNA (25 nM; left hand-side panel), a cocktail of AKT1/AKT2/AKT3 siRNAs (25 nM each; right hand-side panel) or siRNA control (Scramble; 25 nM for MCL1 siRNA comparison and 75 nM for AKT1/2/3 siRNA comparison) in LNCaP95 **(E)** and C4-2 **(F)**. Three biological replicates are shown. Asterisks (*$p < 0.05$; **$p < 0.01$; ***$p < 0.001$) indicate statistically significant differences between groups.

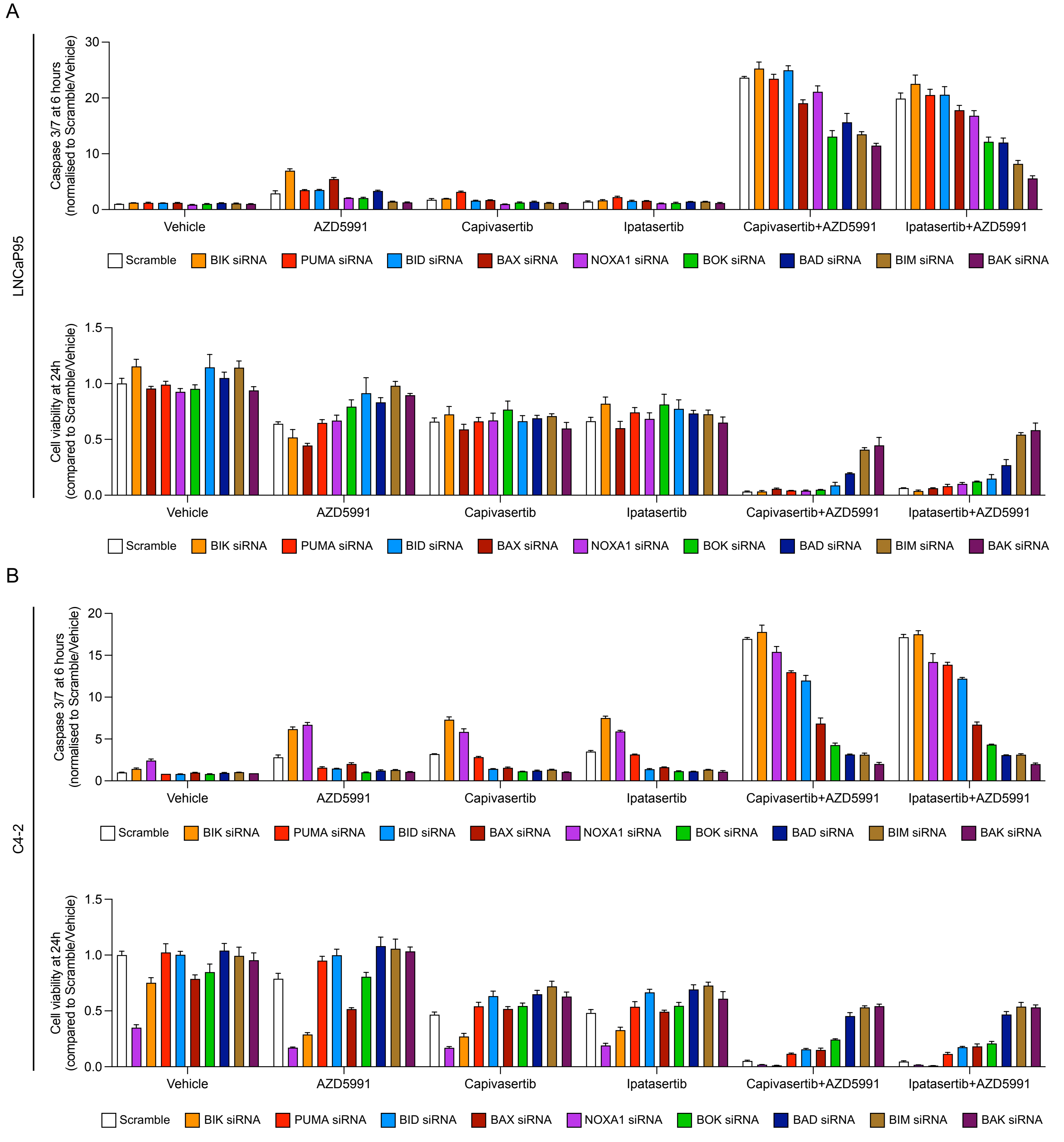

Supplementary Figure 14. Pro-apoptotic BCL2 family siRNA screen in CRPC cells. (A-B) Caspase 3/7 (at 6 hours; top panel) levels and cell viability (at 24 hours; bottom panel) in response to AZD5991, capivasertib, ipatasertib and combined treatment, 72 hours after transfection with BH3-only siRNAs (BIK, PUMA, BID, BAX, NOXA1, BOK, BAD, BIM, BAK; 72 hours) in LNCaP95 **(A)** and C4-2 **(B)** cells. Drugs were used at 1 μM and siRNAs at 50 nM. The mean ± standard error of the mean from three biological experiments in technical singlets.

Supplementary Figure 15

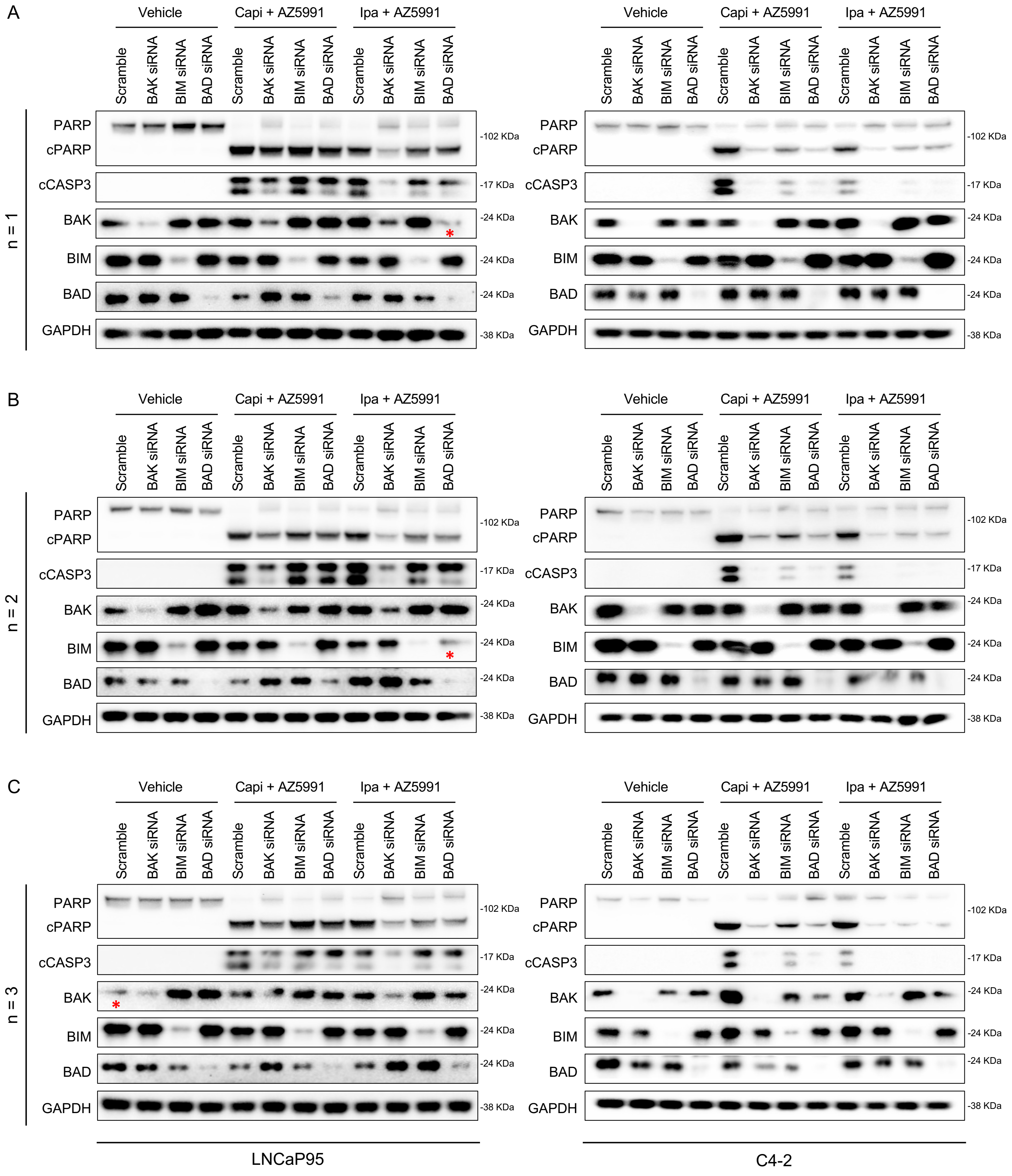

Supplementary Figure 15. Individual BIM, BAD and BAK silencing partially prevents the induction of apoptosis in response to AKT and MCL1 co-inhibition in CRPC cell lines. Protein levels of cleaved PARP, cleaved caspase 3, BAK, BIM and BAD (after 6 hours of treatment) in response to vehicle, capivasertib+AZD5991 and ipatasertib+AZD5991, upon transfection with BAK, BIM, BAD or control siRNA (Scramble) for 72 hours in LNCaP95 (left hand-side panel) and C4-2 (right hand-side panel). Drugs were used at 1 μM and siRNAs at 50 nM. Three biological replicates are shown: n1 **(A)**, n2 **(B)** and n3 **(C)** in technical singlets. Asterisks on the membranes indicate technical problems, likely due to suboptimal transfer at membrane edges.

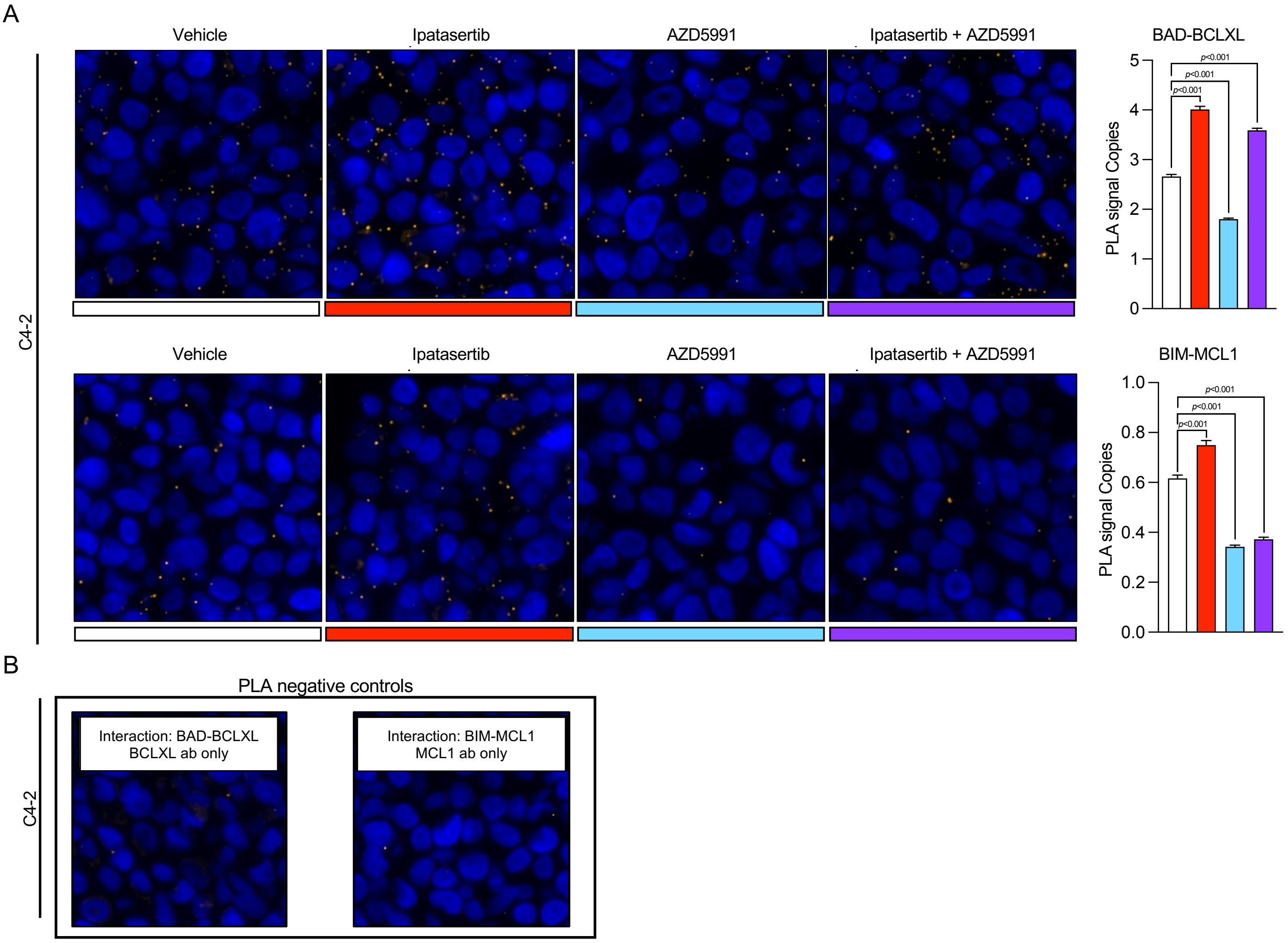

Supplementary Figure 16. Proximity Ligation Assay (PLA) for BAD-BCLXL and BIM-MCL1 Interactions. (A) BAD-BCLXL interaction (top panel) and BIM-MCL1 interaction (bottom panel) in response to Ipatasertib, AZD5991, and combined treatment. Cells were treated with drugs (1 μM) for 72 hours. Signal quantification (number of dots) was calculated with HALO system and is depicted in the right-hand side. The mean ± standard deviation (>1000 cells counted) is shown. One-way ANOVA with post-hoc Tukey was performed. **(B)** Negative controls (BCLXL and MCL1 primary antibodies only) for BAD-BCLXL interaction (left hand-side panel) and BIM-MCL1 interaction (right hand-side panel) in C4-2 cells.

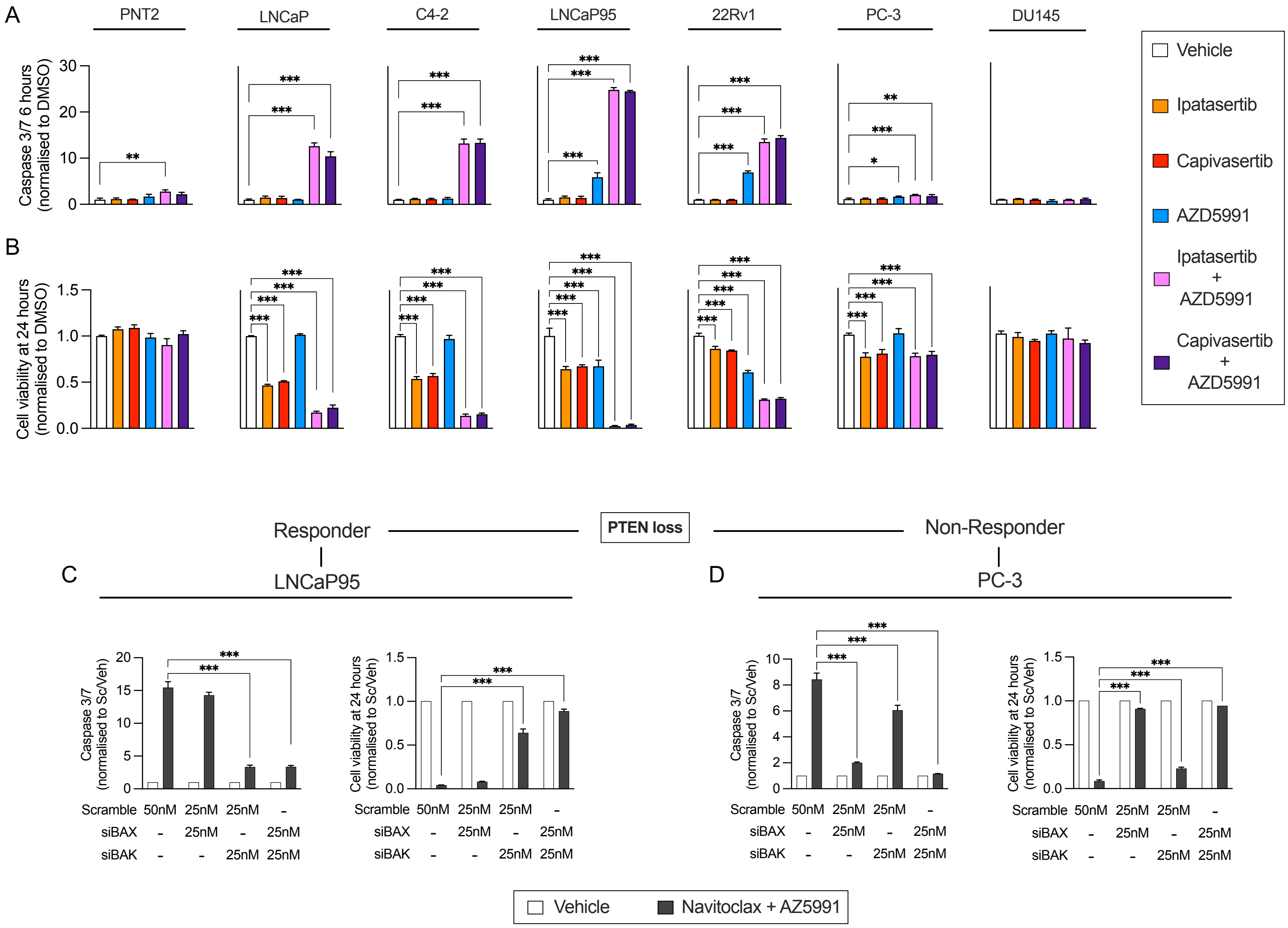

Supplementary Figure 17. AKT and MCL1 co-inhibition drives BAK-dependent apoptosis in prostate cancer cells harbouring PI3K/AKT pathway hyperactivating aberrations. (A) Caspase 3/7 activity (at 6 hours; Caspase-Glo 3/7 2D assay; top panels) and cell viability (at 24 hours; CellTiter-Glo 2D assay; bottom panels) in PNT2, LNCaP, C4-2, LNCaP95, 22Rv1, PC-3 and DU145 cell lines in response to vehicle (DMSO), capivasertib (1 μM), ipatasertib (1 μM), AZD5991 (1 μM) and their combinations. One-way ANOVA with post-hoc Tukey test was performed. (B) Effects of navitoclax (1μM) and AZD5991 (1μM) combined treatment upon knockdown of BAX, BAK, and BAX/BAK (using specific OnTarget siRNAs at 25 nM for 72 hours) or OnTarget Control siRNA (50nM, 72h) on caspase 3/7 activity (6 hours) and cell viability (24 hours) in LNCaP95 (left hand-side panels) and PC-3 (right hand-side panels). One-way ANOVA with post-hoc Tukey test was performed. Asterisks (*$p < 0.05$; **$p < 0.01$; ***$p < 0.001$) indicate statistically significant differences between groups.

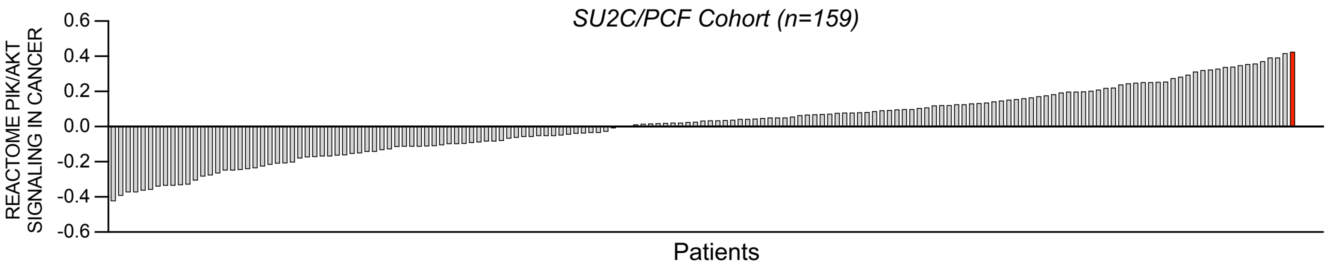

Supplementary Figure 18. PI3K/AKT pathway activity across the SU2C/PCF CRPC cohort patients. Distribution of REACTOME PI3K/AKT signaling in cancer pathway enrichment in patients from SU2C/PCF CRPC cohort. Red bar: Biopsy used for CP50 PDX development.

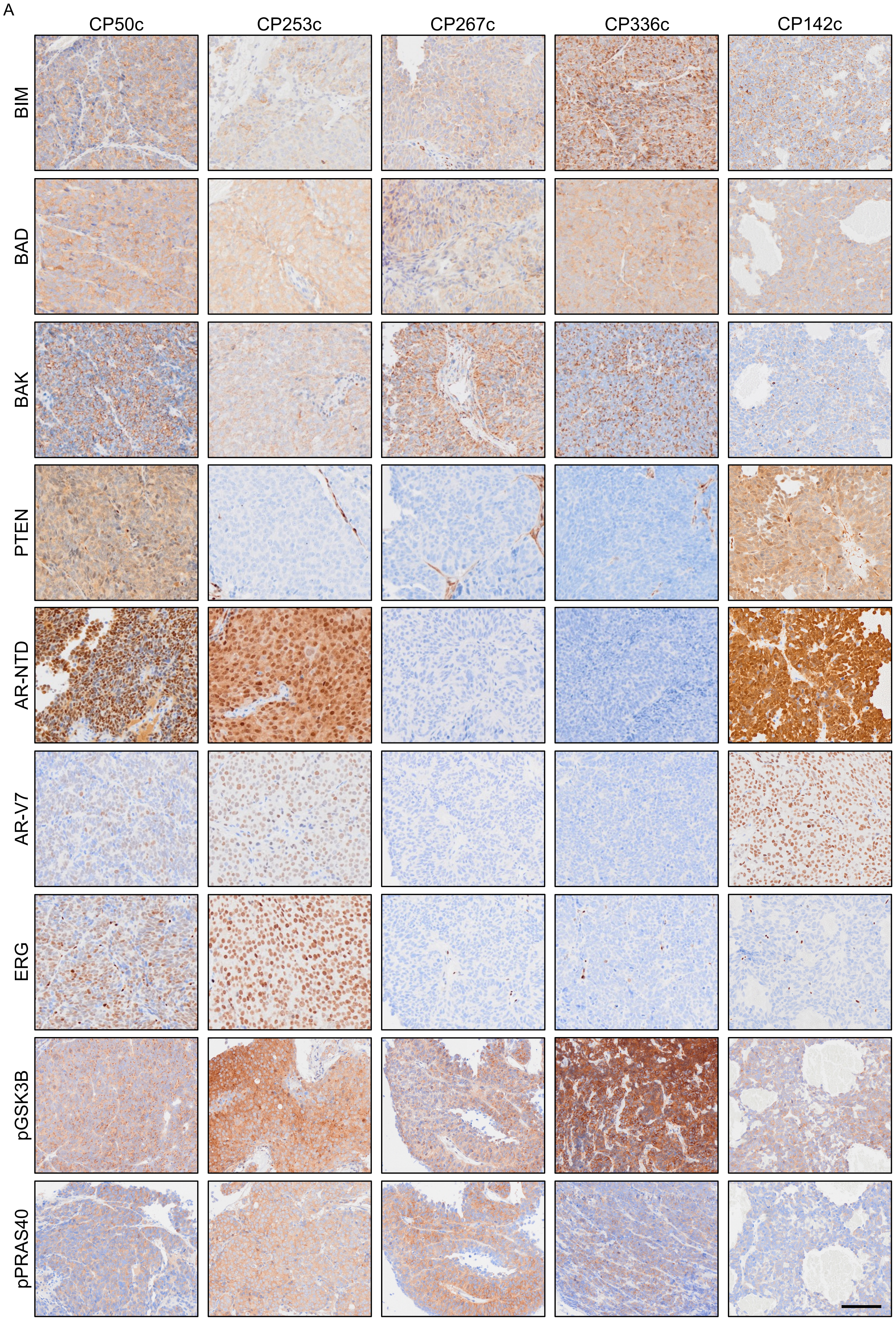
A
CP50c
CP253c
CP267c
CP336c
CP142c
BIM
BAD
BAK
PTEN
AR-NTD
AR-V7
ERG
pGSK3B
pPRAS40

Supplementary Figure 19. Characterisation of CP50c, CP253c, CP267c, CP336c and CP142c CRPC PDXs. Micrographs showing BIM, BAD, BAK, PTEN, AR-NTD, AR-V7, ERG, phospho-GSK3B (Ser9; pGSK3B) and phospho-PRAS40 (Thr246; pPRAS40) protein levels by immunohistochemistry. The scale bar indicates a length of 100 µm.

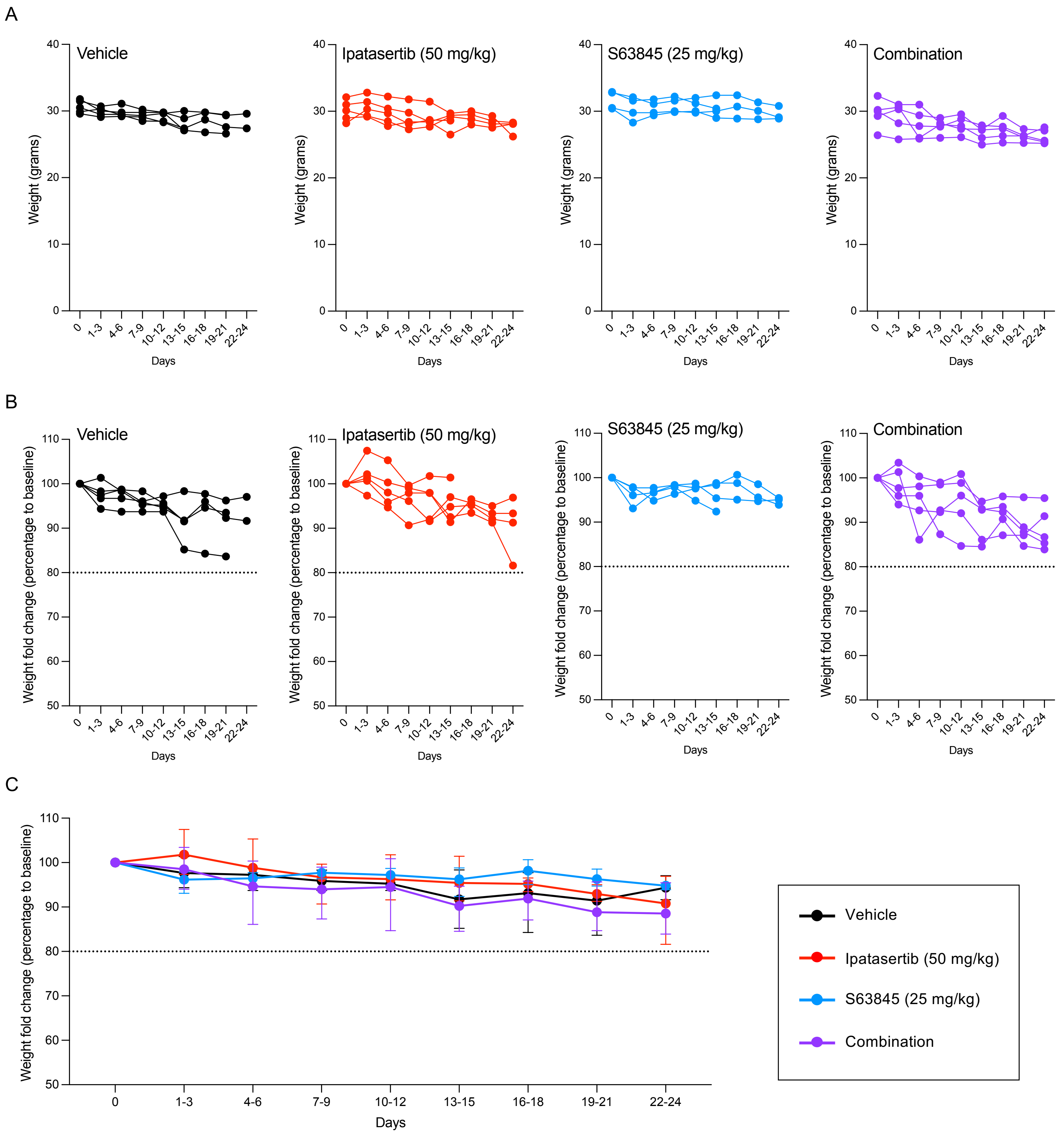

Supplementary Figure 20. Mouse body weights during the in vivo experiment. (A-B) Individual mouse body weights are shown for treatments with vehicle, ipatasertib, S63845, and their combination. Data are presented as raw body weight (in grams) **(A)** and as percentages relative to baseline **(B)**. **(C)** Mean body weight per treatment group, with error bars representing the standard error of the mean (SEM).

Supplementary Figure 21

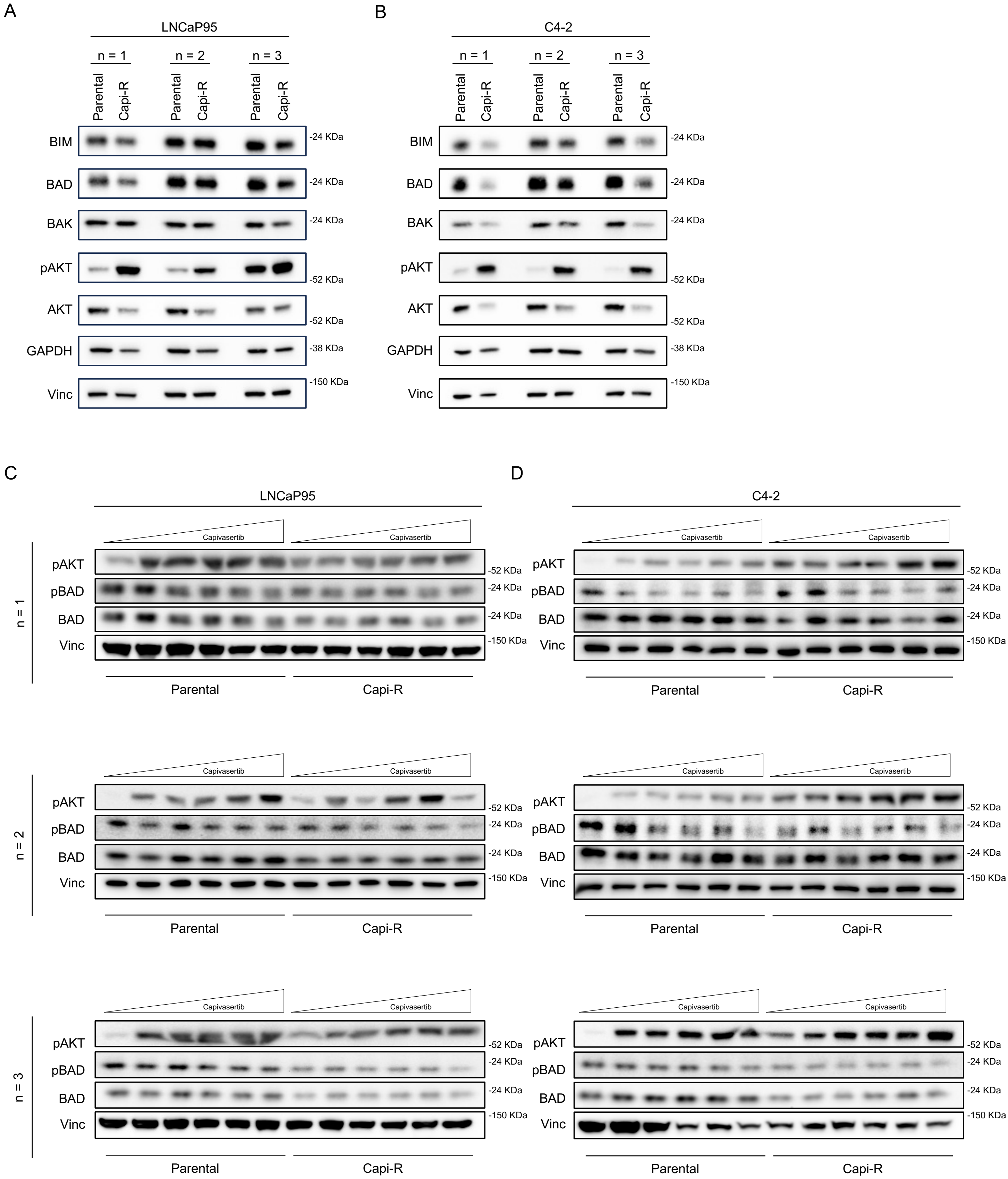

Supplementary Figure 21. (A-B) Comparison of protein levels of BIM, BAD, BAK, total and p-AKT^{Ser473} between parental and Capi-R cells in C4-2 **(A)** and LNCaP95 **(B)**. Vinculin was used as a housekeeping protein. **(C-D)** Protein levels of total and p-BAD^{Ser136}, total BAD and p-AKT^{Ser473} in response to vehicle and varying capivasertib concentrations (0.1 µM 0.5 µM, 1 µM, 5µM) between parental and Capi-R cells in LNCaP95 **(C)** and C4-2 **(D)**. Vinculin was used as a housekeeping protein. All the experiments were performed in three biological triplicates and technical singlets.

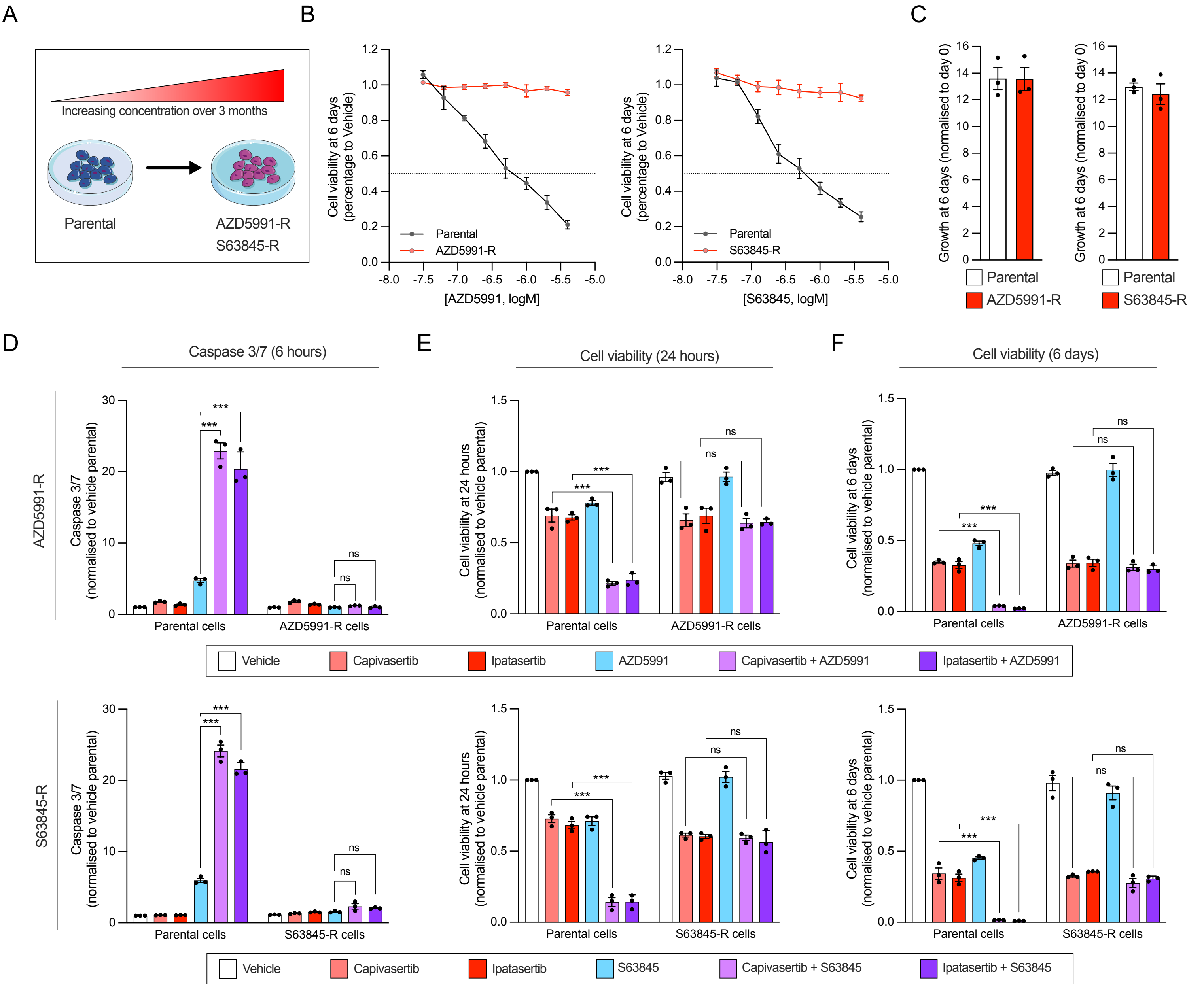

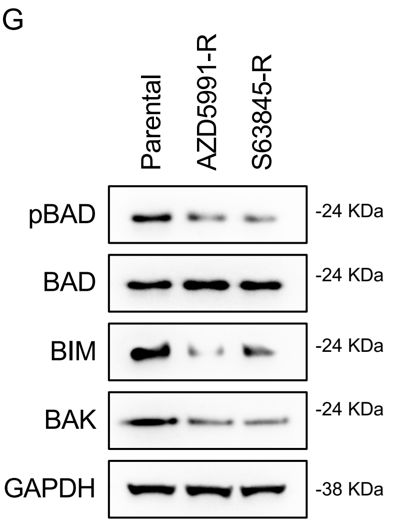

Supplementary Figure 22. Acquired resistance to MCL1 inhibitors abolishes the synergistic effects of AKT and MCL1 co-inhibition. (A) Schematic of how AZD5991- and S63845-resistant LNCaP95 cells were developed. Image adapted from Servier Medical Art (https://smart.servier.com/), licensed under CC BY 4.0 (https://creativecommons.org/licenses/by/4.0/). **(B)** Dose-response curves for AZD5991 (left) and S63845 (right) in AZD5991-resistant (AZD5991-R) and S63845-resistant (S63845-R) vs parental LNCaP95 cells. **(C)** Cell growth at day 6 (fold change from day 0) in AZD5991-R and S63845-R cells compared to parental cells. Statistical analysis was performed using unpaired t-tests. **(D-F)** Caspase 3/7 activity at 6 hours (D), cell viability at 24 hours (E), and 6 days (F) in AZD5991-R (top panel) and S63845-R (bottom panel) vs parental cells treated with vehicle (DMSO), capivasertib (1 μM), ipatasertib (1 μM), AZD5991 (1 μM), or combination treatments. Two-way ANOVA with post-hoc Tukey tests was used for analysis. **(G)** Western blot comparing protein levels of phospho-BAD (Ser136), BAD, BIM, and BAK between parental, AZD5991-R, and S63845-R cells. GAPDH served as a loading control. All experiments were performed in triplicate except the western blot (n=1). Error bars represent the standard error of the mean (SEM). Asterisks (*$p < 0.05$; **$p < 0.01$; ***$p < 0.001$) indicate statistically significant differences between groups.

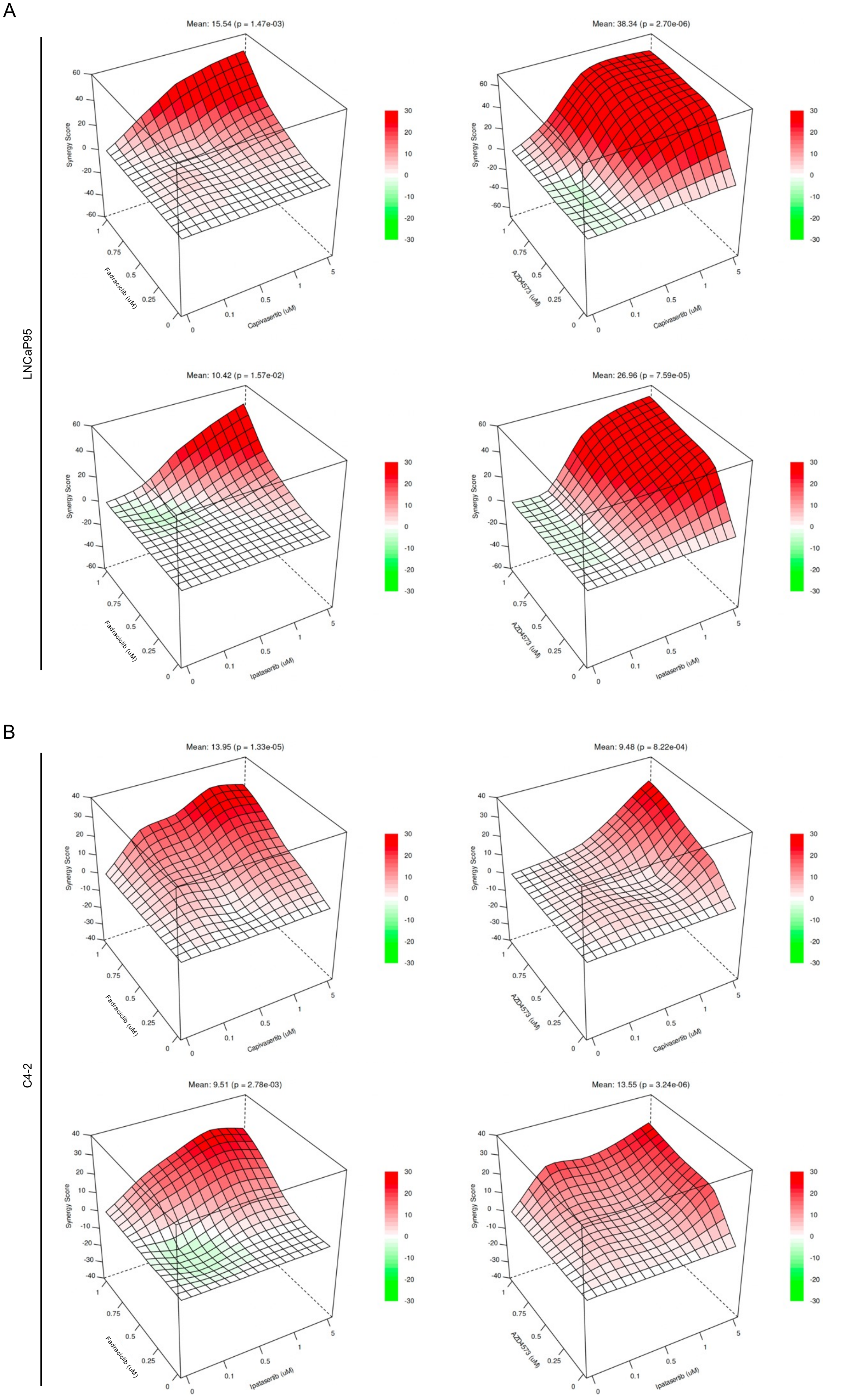

Supplementary Figure 23. AKT inhibitors synergise with CDK9 inhibitors to decrease cell viability in CRPC cell lines. (A-B) Zero Interaction Potency (ZIP) synergy score surface plots of fadraciclib and AZD4573 combined with AKT inhibitors (capivasertib and ipatasertib) in LNCaP95 **(A)** and C4-2 **(B)** cells.

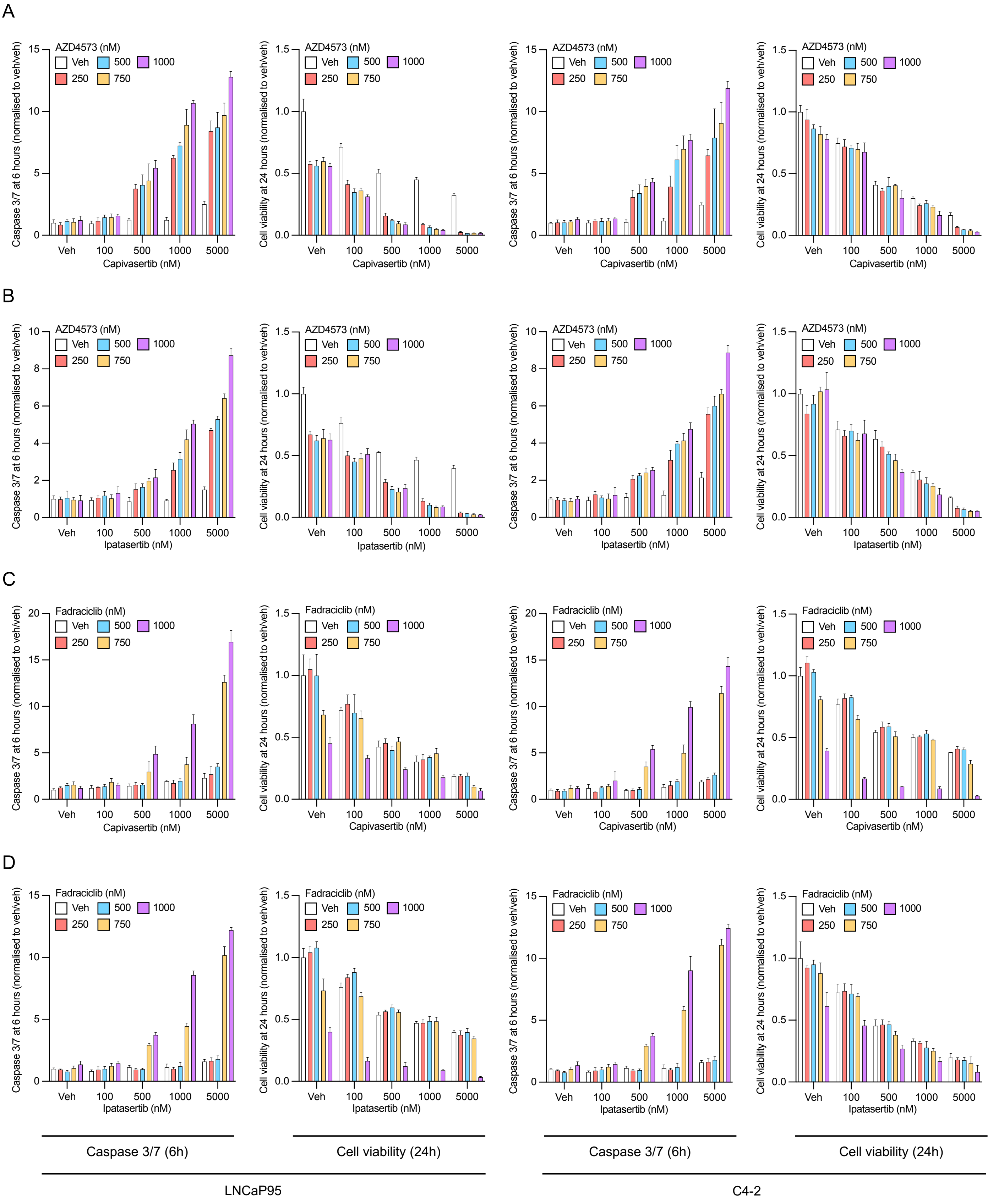

Supplementary Figure 24. Co-inhibition of AKT and CDK9 drives apoptotic cell death in a dose-dependent manner in CRPC cells. (A-D) Caspase 3/7 activity (at 6 hours; Caspase-Glo 3/7 assay) and cell viability (at 24 hours; CellTiter-Glo assay) are shown in response to varying concentrations of AZD4573 (A-B) and fadraciclib **(C-D)** in absence or presence of varying concentrations of capivasertib **(A-C)** and ipatasertib **(B-D)** in LNCaP95 and C4-2 cells. The experiment was performed as a single biological replicate in technical triplicates and the mean ± standard deviation is shown.

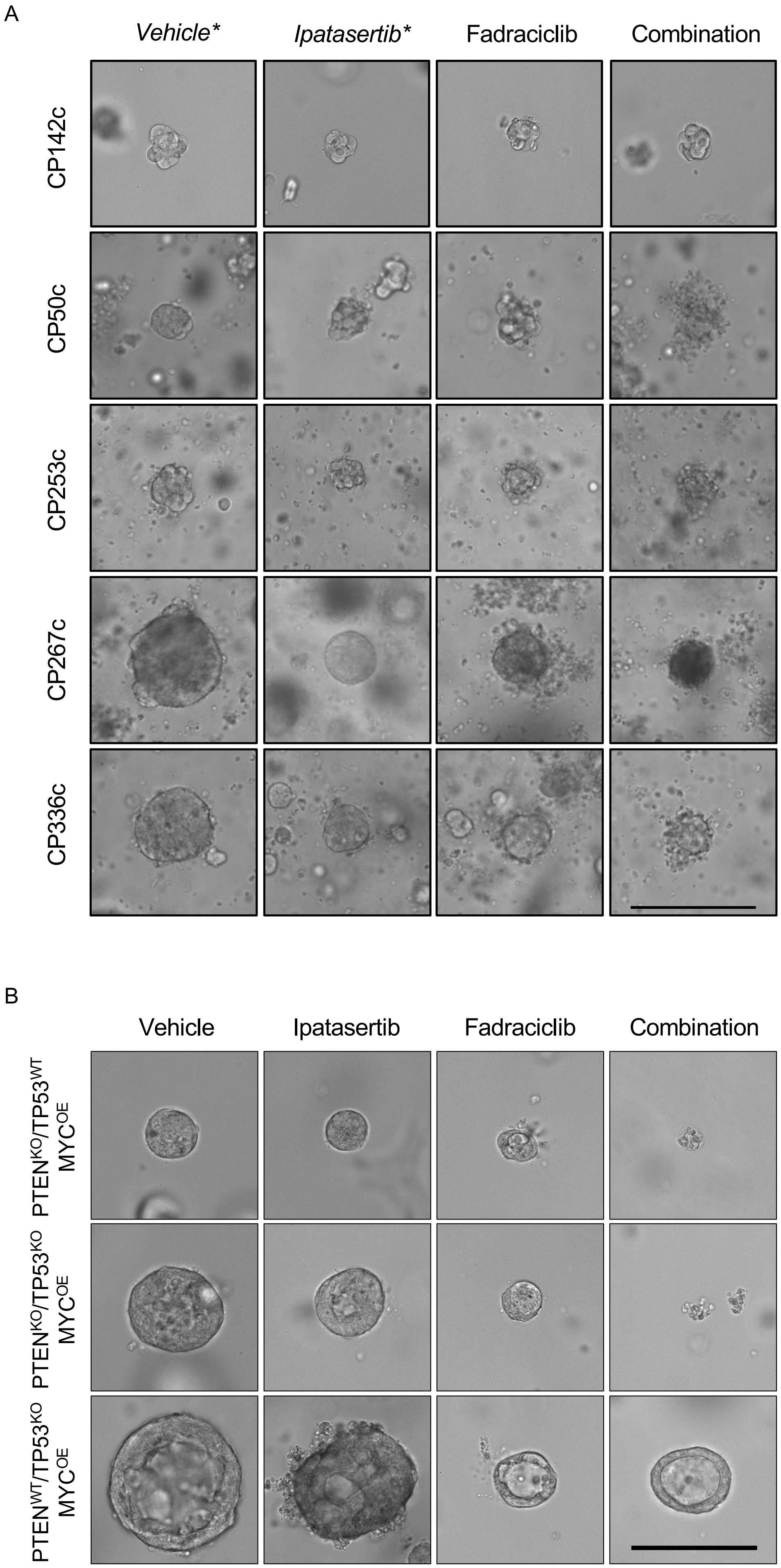

Supplementary Figure 25. Representative microscopy images. (A) PDX-Os treated with ipatasertib, fadraciclib and combined treatment. The vehicle and ipatasertib images are used also in Figure 4E (but CP142c) since those arms were shared for both experiments. **(B)** ProMPt-Os treated with ipatasertib, fadraciclib and combined treatment. Drugs were used at 1μM. Images were taken on day 4 after treatment. The scale bar indicates a length of 200 μm.

Supplementary Figure 26

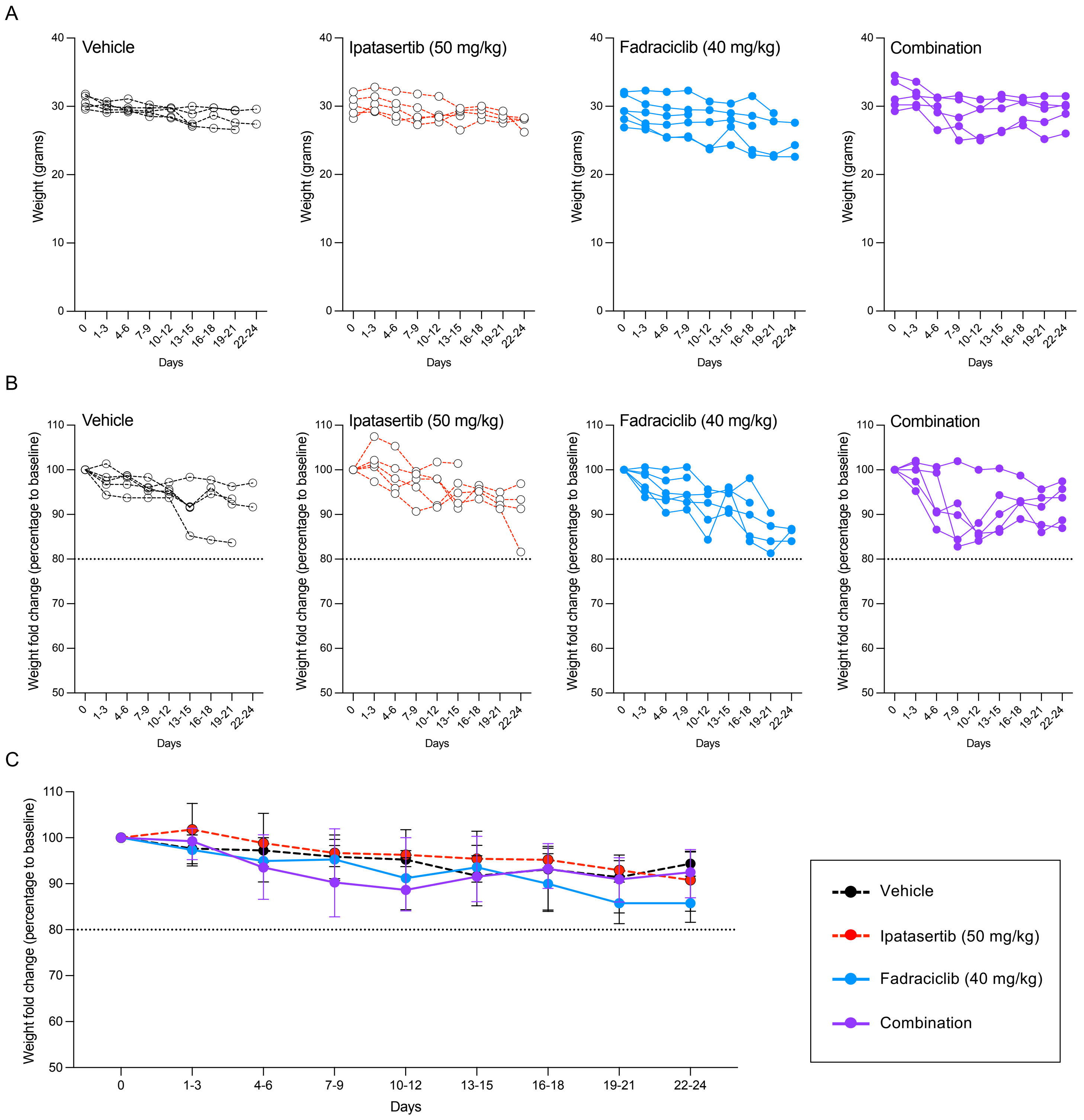

Supplementary Figure 26. Mouse body weights during the in vivo experiment. (A-B) Individual mouse body weights are shown for treatments with vehicle, ipatasertib, fadraciclib, and their combination. Data are presented as raw body weight (in grams) **(A)** and as percentages relative to baseline **(B)**. **(C)** Mean body weight per treatment group, with error bars representing the standard error of the mean (SEM). Vehicle and ipatasertib arms (depicted as dotted lines and clear symbols) have been already shown in Supplementary Figure 20 (same experiment).

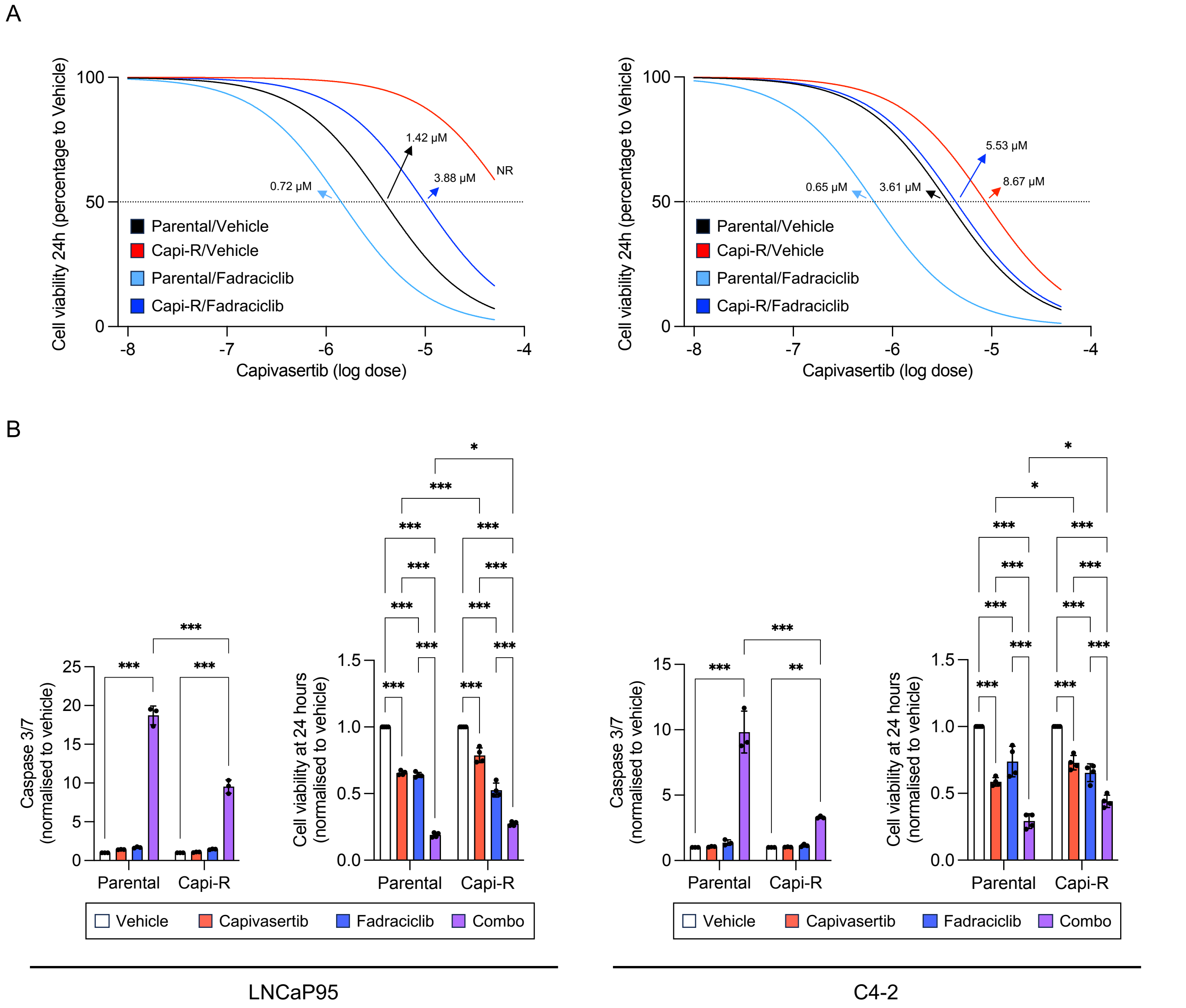

Supplementary Figure 27. CRPC cells with acquired resistance to capivasertib remain sensitive to AKT and CDK9 co-inhibition (A). Comparison of capivasertib dose-response curves between Capi-R and parental LNCaP95 (left hand-side panel) and C4-2 (right hand-side panel) cells in presence and absence of fadraciclib (1 µM). Cell viability was determined using CellTiter-Glo 2D assay (at 24 hours). **(B)** Caspase 3/7 activity (at 6 hours; Caspase-Glo 3/7 2D assay) and cell viability (at 24 hours; CellTiter-Glo 2D assay) in parental and Capi-R LNCaP95 (left hand-side panel) and C4-2 (right hand-side panel) cells treated with vehicle, capivasertib (1 µM), fadraciclib (1 µM) and combined treatment. All the experiments were performed in three biological triplicates and technical triplicates. The standard error of the mean is shown. One-way ANOVA with post-hoc Tukey was performed. Asterisks (* $p < 0.05$; ** $p < 0.01$; *** $p < 0.001$) indicate statistically significant differences between groups. NR: Not reached.

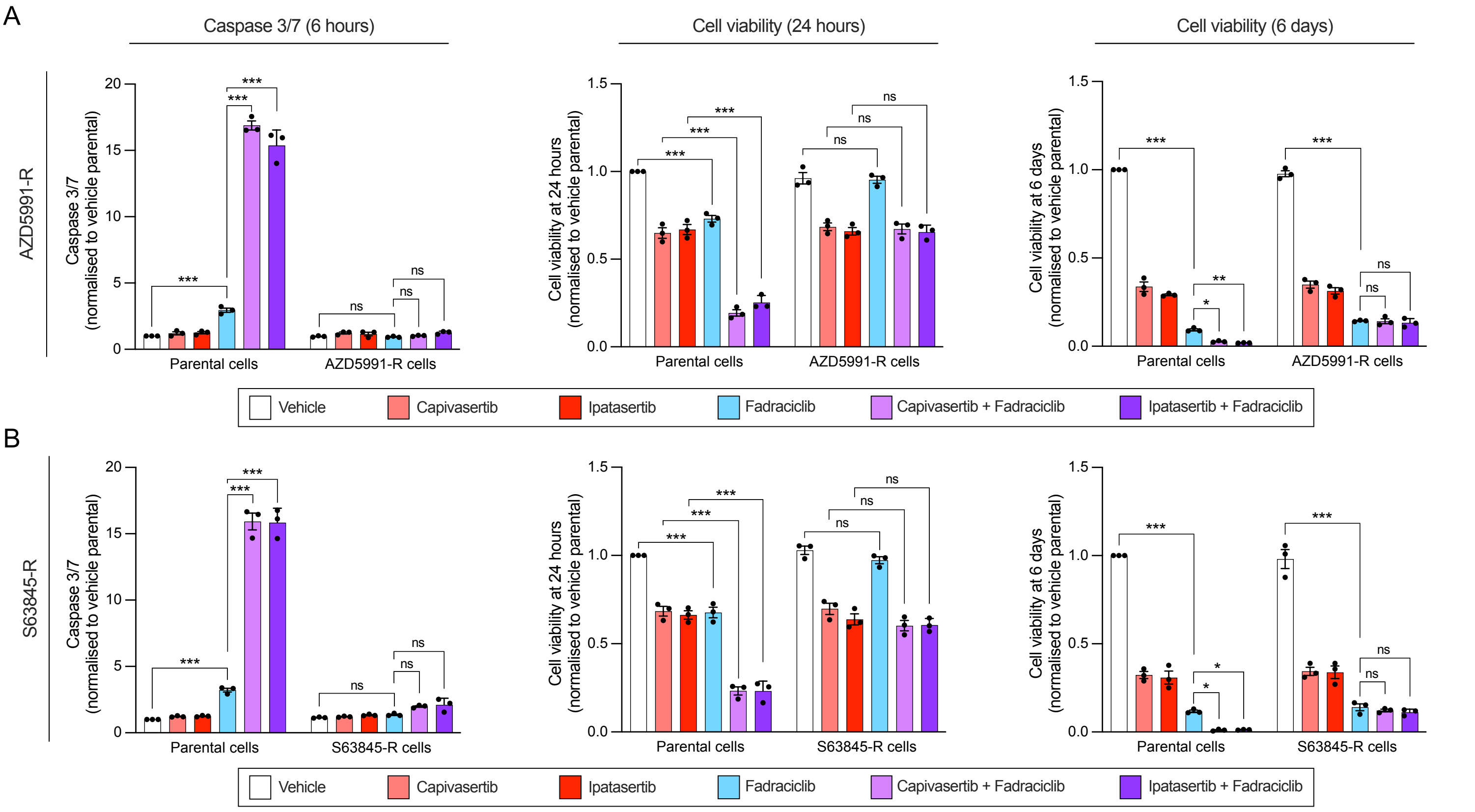

Supplementary Figure 28. Acquired resistance to MCL1 inhibitors abolishes the synergistic effects of AKT and CDK9 co-inhibition. (A-B) Caspase 3/7 activity at 6 hours (left), cell viability at 24 hours (center), and 6 days (right) in AZD5991-R (A) and S63845-R (B) vs parental cells treated with vehicle (DMSO), capivasertib (1 μM), ipatasertib (1 μM), fadraciclib (1 μM), or combination treatments. Two-way ANOVA with post-hoc Tukey tests was used for analysis. All experiments were performed in triplicate. Error bars represent the standard error of the mean (SEM). Statistically significant differences are indicated (*p < 0.05; **p<0.01; ***p < 0.001).

Supplementary tables

	FIRSTANA (N=79)	PROSELICA (N=73)	Overall (N=152)
**ECOG PS >=2**			
Yes	2 (2.5%)	10 (13.7%)	12 (7.9%)
No	77 (97.5%)	63 (86.3%)	140 (92.1%)
**RECIST-Measurable**			
No	33 (41.8%)	35 (47.9%)	68 (44.7%)
Yes	46 (58.2%)	38 (52.1%)	84 (55.3%)
**Visceral Disease**			
No	63 (79.7%)	52 (71.2%)	115 (75.7%)
Yes	16 (20.3%)	21 (28.8%)	37 (24.3%)
**Bone-Only Disease**			
No	58 (73.4%)	50 (68.5%)	108 (71.1%)
Yes	21 (26.6%)	23 (31.5%)	44 (28.9%)
**Pain at Baseline**			
No	13 (16.5%)	14 (19.2%)	27 (17.8%)
Yes	57 (72.2%)	55 (75.3%)	112 (73.7%)
Missing	9 (11.4%)	4 (5.5%)	13 (8.6%)
**Gleason Score < 8**			
No	41 (51.9%)	30 (41.1%)	71 (46.7%)
Yes	34 (43.0%)	37 (50.7%)	71 (46.7%)
Missing	4 (5.1%)	6 (8.2%)	10 (6.6%)
**PSA**			
Median [Q1, Q3]	73.1 [24.0, 207]	173 [96.3, 625]	145 [40.3, 323]
**HB**			
Median [Q1, Q3]	118 [110, 126]	110 [104, 122]	117 [106, 124]
**LDH**			
Median [Q1, Q3]	286 [220, 377]	376 [238, 639]	301 [225, 477]
**ALP**			
Median [Q1, Q3]	158 [96.3, 363]	225 [116, 437]	192 [107, 430]
Missing	1 (1.3%)	0 (0%)	1 (0.7%)
**ALB**			
Median [Q1, Q3]	40.0 [37.4, 42.9]	39.9 [35.8, 42.2]	40.0 [36.9, 42.4]
Missing	3 (3.8%)	1 (1.4%)	4 (2.6%)
**NLR**			

Median [Q1, Q3]	2.36 [1.68, 3.83]	2.65 [1.65, 3.89]	2.53 [1.66, 3.88]
**Prior Abi/Enza Treatment**			
Yes	0 (0%)	26 (35.6%)	26 (17.1%)
No	79 (100%)	47 (64.4%)	126 (82.9%)
**Prior Radical Radiotherapy**			
Yes	14 (17.7%)	21 (28.8%)	35 (23.0%)
No	65 (82.3%)	52 (71.2%)	117 (77.0%)
**Prior Prostatectomy**			
Yes	15 (19.0%)	10 (13.7%)	25 (16.4%)
No	64 (81.0%)	63 (86.3%)	127 (83.6%

Supplementary Table 1. Clinical characteristics of FIRSTANA/PROSELICA cohort.

ECOG PS2: Eastern Cooperative Oncology Group Performance Status 2. RECIST: Response Evaluation Criteria In Solid Tumours. PSA: Prostate Specific Antigen. HB: Haemoglobin. LDH: Lactate Dehydrogenase. ALP: Alkaline Phosphatase. ALB: Albumin. NLR: Neutrophil-to-Lymphocyte Ratio. Abi: Abiraterone. Enza: Enzalutamide.

Protein Target	Supplier/ Catalogue No	Species	Instrument	Retrieval Method	Dilution/ Incubation Time	Detection System	Controls
AR-NTD	Dako, Agilent #M3562	Ms mono	BioGenex i6000 autostainer (Launch Diagnostics)	pH8.1 Tris/EDTA Water bath	1:1000 1 hour	Dako REAL Envision detection system (K4061, Agilent)	VCaP, PC3 cell lines. Prostate. Mouse IgG as negative control.
AR-V7	RevMAb Biosciences #31-1109-00	Rb mono	Bond RX (Leica BIOSYSTEMS)	ER2 10 minutes	1:500 30 minutes	Bond Polymer Refine Detection (DS9800, Leica BIOSYSTEMS)	22Rv1, VCaP and PC3 cell lines. Rabbit IgG as negative control.
BAD	Abcam ab32445	Rb mono	Bond RX (Leica BIOSYSTEMS)	ER2 10 minutes	1:50 15 minutes	Bond Polymer Refine Detection (DS9800, Leica BIOSYSTEMS)	DU145 control/ DU145 siBAD. Kidney. Rabbit IgG as negative control.
BAK	Cell Signaling Technology #12105	Rb mono	BioGenex i6000 autostainer (Launch Diagnostics)	pH8.1 Tris/EDTA Microwave	1:100 1 hour	Dako REAL Envision detection system (K4061, Agilent)	LNCaP95 control/ LNCaP95 siBAK. Prostate, appendix. Rabbit IgG as negative control.
BCLXL	Cell Signaling Technology #2764	Rb mono	Bond RX (Leica BIOSYSTEMS)	ER1 30 minutes	1:1000 15 minutes	Bond Polymer Refine Detection (DS9800, Leica BIOSYSTEMS)	DU145 control/ DU145 siBCLXL. Kidney. Rabbit IgG as negative control.
BIM	Cell Signaling Technology #2933	Rb mono	Bond RX (Leica BIOSYSTEMS)	ER2 10 minutes	1:100 15 minutes	Bond Polymer Refine Detection (DS9800, Leica BIOSYSTEMS)	LNCaP95 control/ LNCaP95 siBIM. Appendix, bone marrow. Rabbit IgG as negative control.
Cleaved Caspase 3	Cell Signaling Technology #9661	Rb poly	BioGenex i6000 autostainer (Launch Diagnostics)	pH6 citrate Microwave	1:400 1 hour	VECTASTAIN Elite ABC-HRP Kit (PK-6101, 2BScientific)	Appendix. Rabbit IgG as negative control.
ERG	Abcam ab92513	Rb mono	Bond RX (Leica BIOSYSTEMS)	ER1 30 minutes	1:250 15 minutes	Bond Polymer Refine Detection (DS9800, Leica BIOSYSTEMS)	VCaP, PC3 cell lines. Prostate. Rabbit IgG as negative control.
KI67	Dako, Agilent #M7240	Ms mono	Bond RX (Leica BIOSYSTEMS)	ER1 30 minutes	1:100 30 minutes	Bond Polymer Refine Detection (DS9800, Leica BIOSYSTEMS)	Appendix. Mouse IgG as negative control.

MCL1	Proteintech #16225-1-AP	Rb poly	BioGenex i6000 autostainer (Launch Diagnostics)	pH6 citrate Pressure cooker	1:500 1 hour	Dako REAL Envision detection system (K4061, Agilent)	DU145 control/DU145 siMCL1. Prostate, placenta. Rabbit IgG as negative control.
pGSK-3β	Cell Signaling Technology #9323	Rb mono	BioGenex i6000 autostainer (Launch Diagnostics)	pH6 citrate Microwave	1:50 1 hour	VECTASTAIN Elite ABC-HRP Kit (PK-6101, 2BScientific)	LNCaP, PC3. Appendix. Rabbit IgG as negative control.
pPRAS40	Cell Signaling Technology #13175	Rb mono	BioGenex i6000 autostainer (Launch Diagnostics)	pH6 citrate Microwave	1:400 1 hour	VECTASTAIN Elite ABC-HRP Kit (PK-6101, 2BScientific)	22Rv1, PC3. Prostate. Rabbit IgG as negative control.
PTEN	Cell Signaling Technology #9188	Rb mono	BioGenex i6000 autostainer (Launch Diagnostics)	pH6 citrate Microwave	1:250 1 hour	VECTASTAIN Elite ABC-HRP Kit (PK-6101, 2BScientific)	22Rv1, PC3 cell lines. Rabbit IgG as negative control.

Supplementary Table 2. Primary antibodies and protocols for immunohistochemistry.

Rb: Rabbit. Ms: Mouse. Mono: Monoclonal. Poly: Polyclonal. ABC-HRP: Avidin-Biotin Complex-Horseradish Peroxidase. ER: Epitope Retrieval. The Dako REAL Envision detection system (K4061, Agilent) has been discontinued.

Cell line	Supplier	Catalogue number	Media	Serum
PNT2	Sigma-Aldrich	95012613	RPMI	FBS
LNCaP	ATCC	CRL-1740	RPMI	FBS
C4-2	ATCC	CRL-3314	DMEM	FBS
LNCaP95	Dr Meeker/Dr Luo*	NA	RPMI[#]	CSS
22Rv1	ATCC	CRL-2505	RPMI	FBS
DU145	ATCC	HTB-81	DMEM	FBS
PC3	ATCC	CRL-1345	RPMI	FBS

Supplementary Table 3. Cell lines.

ATCC – American type culture collection, FBS – fetal bovine serum, CSS – charcoal stripped serum, * - LNCaP95 cells were kindly provided by Drs. Alan K Meeker and Jun Luo (Johns Hopkins University, Baltimore, Maryland, USA), # phenol red free.

Methotrexate	Megestrol acetate	Mitoxantrone	Imatinib	Ixazomib citrate	Entrectinib
Busulfan	Trifluridine	Amifostine	Lapatinib	Ponatinib	Pomalidomide
Thioguanine	Procarbazine hydrochloride	Fludarabine phosphate	Nilotinib	Belinostat	Glasdegib
Mercaptopurine	Lomustine	Temozolomide	Sorafenib	Idelalisib	Ceritinib
Mechlorethamine hydrochloride	Daunorubicin hydrochloride	Imiquimod	Lenalidomide	Vandetanib	Tazemetostat
Allopurinol	Streptozocin	Carmustine	Ixabepilone	Cabozantinib	Capmatinib
Dactinomycin	Arsenic trioxide	Clofarabine	Raloxifene	Panobinostat	Encorafenib
Chlorambucil	Azacitidine	Vinorelbine tartrate	Abiraterone	Erismodegib	Ribociclib
Thiotepa	Cladribine	Topotecan hydrochloride	Sunitinib	Plerixafor	Osimertinib
Melphalan hydrochloride	Ifosfamide	Gemcitabine hydrochloride	Afatinib	Vemurafenib	Lorlatinib
Triethylenemelamine	Cisplatin	Irinotecan hydrochloride	Olaparib	Cabazitaxel	Selinexor
Altretamine	Tretinoin	Docetaxel	Romidepsin	Ibrutinib	Erdafitinib
Aminolevulinic acid hydrochloride	Teniposide	Temsirolimus	Pralatrexate	Ruxolitinib	Larotrectinib
Fluorouracil	Doxorubicin hydrochloride	Vorinostat	Niraparib hydrochloride	Regorafenib	Brigatinib
Plicamycin	Bleomycin sulfate	Estramustine phosphate sodium	Pemetrexed, Disodium salt, Heptahydrate	Alectinib	Gilteritinib
Pipobroman	Paclitaxel	Capecitabine	Enzalutamide	Binimetinib	Enasidenib
Cyclophosphamide	Decitabine	Exemestane	Lenvatinib	Dabrafenib mesylate	Uridine triacetate
Mitomycin	Bendamustine hydrochloride	Gefitinib	Nelarabine	ARRY-380	Ivosidenib
Floxuridine	Etoposide	Erlotinib hydrochloride	Vismodegib	Bosutinib	Pexidartinib
Hydroxyurea	Dexrazoxane	Fulvestrant	Rucaparib phosphate	Dacomitinib	Acalabrutinib
Uracil mustard	Tamoxifen citrate	Anastrozole	Crizotinib	Alpelisib	Avapritinib
Mitotane	Pentostatin	Letrozole	Bortezomib	Venetoclax	Copanlisib tris-HCl
Dacarbazine	Sirolimus	Celecoxib	Neratinib	Talazoparib	Pemigatinib
Methoxsalen	Carboplatin	Zoledronic acid	Axitinib	Fedratinib	Selpercatinib

Vinblastine sulfate	Valrubicin	Dasatinib	Trametinib	Cobimetinib	Zanubrutinib
Cytarabine hydrochloride	Idarubicin hydrochloride	Everolimus	Palbociclib	Abemaciclib	Darolutamide
Thalidomide	Epirubicin hydrochloride	Pazopanib hydrochloride	Carfilzomib	Apalutamide	
Vincristine sulfate	Oxaliplatin	Selumetinib	Omacetaxine mepesuccinate	Duvelisib	

Supplementary Table 4. NCI/NIH FDA-approved anti-cancer "Approved Oncology Drugs Set"

**Gene target**	**Catalogue ID**	**Supplier**
Control	D-001810-10	Dharmacon (Horizon)
AKT1	L-003000-00	
AKT2	L-003001-00	
AKT3	L-003002-00	
MCL1	L-004501-00	
BAD	L-003870-00	
BAK1	L-003305-00	
BAX	L-003308-01	
BID	L-004387-00	
BIK	L-004388-00	
BIM	L-004383-00	
BOK	L-004394-00	
NOXA1	L-026087-01	
PUMA	L-004380-00	

Supplementary Table 5. ON-TARGETplus siRNA pools for gene expression knockdown.

Protein Target	Supplier	Catalogue No.	Species	Dilution
MCL1	Proteintech	16225-1-AP	Rabbit polyclonal	1:5000
BCLXL	Cell Signaling Technology	2764	Rabbit monoclonal	1:1000
Phospho-Bad (Ser136)	Cell Signaling Technology	4366	Rabbit monoclonal	1:250
BAD	Abcam	ab32445	Rabbit monoclonal	1:1000
BIM	Cell Signaling Technology	2933	Rabbit monoclonal	1:1000
BAK	Cell Signaling Technology	12105	Rabbit monoclonal	1:1000
AR-NTD	Dako, Agilent	M3562	Mouse monoclonal	1:1000
KI67	Dako, Agilent	M7240	Mouse monoclonal	1:1000
PARP	Cell Signaling Technology	9542	Rabbit polyclonal	1:1000
Cleaved Caspase3	Cell Signaling Technology	9661	Rabbit polyclonal	1:500
Cleaved Caspase 7	Cell Signaling Technology	9491	Rabbit polyclonal	1:500
Phospho-PRAS40 (Thr246)	Cell Signaling Technology	13175	Rabbit monoclonal	1:1000
Phospho-GSK3β (Ser9)	Cell Signaling Technology	9323	Rabbit monoclonal	1:1000
Phospho-AKT (Ser473)	Cell Signaling Technology	9271	Rabbit polyclonal	1:1000
AKT (pan)	Cell Signaling Technology	4691	Rabbit monoclonal	1:1000
GAPDH	Santa Cruz Biotechnology	sc-32233	Mouse monoclonal	1:2500
Vinculin	Santa Cruz Biotechnology	sc-5286	Mouse monoclonal	1:2500

Supplementary Table 6. Antibodies used for western blot.
