# Supplementary material for: Elucidating molecularly stratified single agent, and combination, therapeutic strategies targeting MCL1 for lethal prostate cancer
Source: Nat Commun. 2025 Oct 8;16:8806. doi: 10.1038/s41467-025-64042-5 (PMC12508096; doi:10.1038/s41467-025-64042-5)
Supplement: Supplementary file 2 — Reporting Summary [file 41467_2025_64042_MOESM2_ESM.pdf]

nature portfolio

Corresponding author(s): Adam Sharp

Last updated by author(s): Jul 17, 2025

Reporting Summary

Nature Portfolio wishes to improve the reproducibility of the work that we publish. This form provides structure for consistency and transparency in reporting. For further information on Nature Portfolio policies, see our Editorial Policies and the Editorial Policy Checklist.

Statistics

For all statistical analyses, confirm that the following items are present in the figure legend, table legend, main text, or Methods section.

n/a	Confirmed	
☐	☒	The exact sample size (n) for each experimental group/condition, given as a discrete number and unit of measurement
☐	☒	A statement on whether measurements were taken from distinct samples or whether the same sample was measured repeatedly
☐	☒	The statistical test(s) used AND whether they are one- or two-sided<br>*Only common tests should be described solely by name; describe more complex techniques in the Methods section.*
☒	☐	A description of all covariates tested
☐	☒	A description of any assumptions or corrections, such as tests of normality and adjustment for multiple comparisons
☐	☒	A full description of the statistical parameters including central tendency (e.g. means) or other basic estimates (e.g. regression coefficient) AND variation (e.g. standard deviation) or associated estimates of uncertainty (e.g. confidence intervals)
☐	☒	For null hypothesis testing, the test statistic (e.g. F, t, r) with confidence intervals, effect sizes, degrees of freedom and P value noted<br>*Give P values as exact values whenever suitable.*
☒	☐	For Bayesian analysis, information on the choice of priors and Markov chain Monte Carlo settings
☒	☐	For hierarchical and complex designs, identification of the appropriate level for tests and full reporting of outcomes
☐	☒	Estimates of effect sizes (e.g. Cohen's d, Pearson's r), indicating how they were calculated

Our web collection on statistics for biologists contains articles on many of the points above.

Software and code

Policy information about availability of computer code

Data collection: No software was used for data collection

Data analysis:

Copy number analysis

RMH and SU2C/PCF cohorts
- Exome-sequencing reads alignment: BWA MEM
- Germline variance and somatic variance: GATK and Mutect2
- Copy number analysis: ASCAT

FIRSTANA/PROSELICA cohorts
- Sequencing reads alignment: BWA MEM
- Aligned reads quantification: HMM copy readCounter
- Copy number and tumour fraction calculation: ichorCNA

National Cancer Institute neoadjuvant cohort
- Exome sequencing of tumour foci and processing of data to derive gene-level somatic copy number estimates was described previously (PMID: 33785256)

TCGA
- CNA segment data was downloaded from cBioportal (PMID: 22588877)

Transcriptomics analyses

RMH and SU2C/PCF cohorts
- Paired-end transcriptome sequencing reads alignment: Tophat2
- Fragments Per Kilobase of transcript per Million mapped reads (FPKM) calculation: Cufflinks
- Pathway analysis: GSEA software
UW/FH cohort
- Sequencing reads were mapping: STAR.v2.7.3a
- Gene level abundance quantification and log2 FPKM transformation: GenomicAlignments and edgeR
TCGA
- Transcriptomic data were downloaded from cBioPortal (PMID: 22588877)

All in vitro and in vivo derived experiment analyses were performed using R Statistical Software version 4.1.3 (R Core Team, Vienna, Austria) or GraphPad Prism version 10 (GraphPad Software Inc., San Diego, California, USA).

For manuscripts utilizing custom algorithms or software that are central to the research but not yet described in published literature, software must be made available to editors and reviewers. We strongly encourage code deposition in a community repository (e.g. GitHub). See the Nature Portfolio guidelines for submitting code & software for further information.

Data

Policy information about availability of data

All manuscripts must include a data availability statement. This statement should provide the following information, where applicable:
- Accession codes, unique identifiers, or web links for publicly available datasets
- A description of any restrictions on data availability
- For clinical datasets or third party data, please ensure that the statement adheres to our policy

Source Data files are provided with this paper. All raw data are available in the Source Data. Transcriptomic and copy number variation datasets used in this study have been previously published and made available (Refs 18, 44, 61, 64-67). No new omics datasets were generated. Further data access requests can be submitted to the corresponding authors. Request must provide clinically relevant rationale and will be reviewed by corresponding authors to determine if the request is subject to any ethical and/or confidentiality considerations. Patient identifier or information that may reveal the patient's identity will not be shared owing to patient confidentiality. Any data or material that can be shared will be done via material transfer agreement with The ICR.

Research involving human participants, their data, or biological material

Policy information about studies with human participants or human data. See also policy information about sex, gender (identity/presentation), and sexual orientation and race, ethnicity and racism.

Reporting on sex and gender	This study includes samples exclusively from prostate cancer patients; therefore, all participants were of male sex. Gender data was not collected from any of the patients.
Reporting on race, ethnicity, or other socially relevant groupings	This study does not involve analyses with or use the constructs of race and/or ethnicity.
Population characteristics	This study does not include age-related information. Other patient characteristics, such as the treatments they were receiving, were available and previously collected in accordance with already approved study protocols.
Recruitment	No patients were recruited for this study. Patient-derived data (RNA and DNA) and samples (for IHC) were already available in our laboratory and were obtained through previously approved study protocols.
Ethics oversight	All samples were obtained from patients enrolled in protocols approved by ethics review committees. All patients provided informed consent. Detailed information can be found in the Methods section of the manuscript.

Note that full information on the approval of the study protocol must also be provided in the manuscript.

Field-specific reporting

Please select the one below that is the best fit for your research. If you are not sure, read the appropriate sections before making your selection.

[x] Life sciences   [ ] Behavioural & social sciences   [ ] Ecological, evolutionary & environmental sciences

For a reference copy of the document with all sections, see nature.com/documents/nr-reporting-summary-flat.pdf

Life sciences study design

All studies must disclose on these points even when the disclosure is negative.

Sample size	The sample size for patient-derived samples (genomic, transcriptomic, and immunohistochemistry) was determined pragmatically, based on the availability of samples and the size of existing datasets. For in vitro assays, three biological replicates were performed. Basal protein level assessments in cell lines, PDX-Os, ProMPt-Os, and co-IP experiments were conducted as biological singlets. For in vivo experiments, sample sizes were selected to ensure sufficient power to achieve statistical significance for the primary endpoint (combination therapy vs. vehicle). The following group sizes were used: vehicle arm (5 mice), ipatasertib arm (5 mice), S63845 arm (4 mice), fadraciclib arm (5 mice), ipatasertib + S63845 arm (5 mice), and ipatasertib + fadraciclib arm (5 mice).

Data exclusions	No data was excluded.
Replication	The experiments were reproducible, as demonstrated by the three biological replicates. Additionally, four independent cohorts were analyzed to validate the genomic and transcriptomic results.
Randomization	Randomization was applied to the assignment of mice to the treatment groups. This was not applicable to the other experiments, which utilized well-studied and characterized models (randomization not necessary).
Blinding	Protein quantification (Immunohistochemistry) was determined by a histopathologist (B.G) blinded to clinical data, or experimental conditions. This was not applicable to the other experiments, which utilized well-studied and characterized models (randomization not necessary).

Reporting for specific materials, systems and methods

We require information from authors about some types of materials, experimental systems and methods used in many studies. Here, indicate whether each material, system or method listed is relevant to your study. If you are not sure if a list item applies to your research, read the appropriate section before selecting a response.

Materials & experimental systems

n/a	Involved in the study
☐	☒ Antibodies
☐	☒ Eukaryotic cell lines
☒	☐ Palaeontology and archaeology
☐	☒ Animals and other organisms
☒	☐ Clinical data
☒	☐ Dual use research of concern
☒	☐ Plants

Methods

n/a	Involved in the study
☒	☐ ChIP-seq
☒	☐ Flow cytometry
☒	☐ MRI-based neuroimaging

Antibodies

Antibodies used

Immunohistochemistry
Listed in order of marker, vendor and cat number
AR-NTD, Dako Agilent, M3562
AR-V7, RevMAb Biosciences, 31-1109-00
BAD, Abcam, ab32445
BAK, Cell Signaling Technology, 12105
BCLXL, Cell Signaling Technology, 2764
BIM, Cell Signaling Technology, 2933
Cleaved Caspase 3, Cell Signaling Technology, 9661
ERG, Abcam, ab92513
KI67, Dako Agilent, M7240
MCL1, Proteintech, 16225-1-AP
pGSK-3β, Cell Signaling Technology, 9323
pPRAS40, Cell Signaling Technology, 13175
PTEN, Cell Signaling Technology, 9188

Western Blot
Listed in order of marker, vendor and cat number
MCL1, Proteintech, 16225-1-AP,
BCLXL, Cell Signaling Technology, 2764
Phospho-Bad (Ser136), Cell Signaling Technology, 4366
BAD, Abcam, ab32445
BIM, Cell Signaling Technology, 2933
BAK, Cell Signaling Technology, 12105
AR-NTD, Dako Agilent, M3562
KI67, Dako Agilent, M7240
PARP, Cell Signaling Technology, 9542
Cleaved Caspase 3, Cell Signaling Technology, 9661
Cleaved Caspase 7, Cell Signaling Technology, 9491
Phospho-PRAS40 (Thr246), Cell Signaling Technology, 13175
Phospho-GSK3β (Ser9), Cell Signaling Technology, 9323
Phospho-AKT (Ser473), Cell Signaling Technology, 9271
AKT (pan), Cell Signaling Technology, 4691
GAPDH, Santa Cruz Biotechnology, sc-32233
Vinculin, Santa Cruz Biotechnology, sc-5286

	Immunoprecipitation<br>Listed in order of marker, vendor and cat number<br>BCLXL, Cell Signaling, 2764<br>MCL1, Proteintech, 16225-1-AP<br>BIM , Cell Signaling Technology, 2933<br><br>Proximity ligation assay<br>Listed in order of marker, vendor, cat number, clone, lot number<br>BAD, abcam, ab32445<br>BCLXL, abcam, ab77571,<br>BIM, Cell Signaling Technology, 2933<br>MCL1, Santa Cruz, sc-69838
Validation	The antibodies used in this study were validated by western blot, comparing protein expression in whole-cell lysates treated with either non-targeting control siRNA or ON-TARGETplus pooled siRNA against the target protein (Dharmacon), as well as using positive and negative control cell lines. Antibodies for IHC were validated by western blot, ensuring detection of a single band and a reduction in signal upon siRNA-mediated knockdown of the target protein (Dharmacon). Validation was further confirmed by IHC using paired cell pellet samples, demonstrating reduced signal in siRNA-treated cells.<br><br>Antibodies for AR-NTD, AR-V7, BCLXL, MCL1, ERG, KI67, pGSK3B, pPRAS40, and PTEN had been validated previously for IHC, while validation of BIM, BAD, and BAK is shown in this study (Supplementary Figures 1 and 2). IgG was used as a control for co-IP, and the omission of one antibody from the interaction was used as a negative control for PLA.<br><br>The full list of antibodies, working dilutions, and incubation times are detailed in the Methods section and Supplementary Tables 2 and 6.

Eukaryotic cell lines

Policy information about cell lines and Sex and Gender in Research

Cell line source(s)	Listed in order of cell line, supplier, cat number, and sex<br>PNT2, Sigma-Aldrich, 95012613, male<br>LNCaP, ATCC, CRL-1740, male<br>C4-2, ATCC, CRL-3314, male<br>22Rv1, ATCC, CRL-2505, male<br>DU145, ATCC, HTB-81, male<br>PC3, ATCC, CRL-1345, male<br>LNCaP95 cells were kindly provided by Drs. Alan K Meeker and Jun Luo (Johns Hopkins University, Baltimore, Maryland, USA)
Authentication	STR profiling confirmed cell lines identity
Mycoplasma contamination	All cell lines tested negative for mycoplasma
Commonly misidentified lines<br>(See ICLAC register)	No commonly misidentified lines were used

Animals and other research organisms

Policy information about studies involving animals; ARRIVE guidelines recommended for reporting animal research, and Sex and Gender in Research

Laboratory animals	PDX tumours were implanted into the left flank of 31 male NOD.Cg-Prkdc scid Il2rg tm1Wjl /SzJ mice (7 weeks/old)
Wild animals	The study did not involve wild animals.
Reporting on sex	The study investigated the response of prostate cancer models; therefore, only male mice were used.
Field-collected samples	The study did not involve samples collected from the field.
Ethics oversight	All procedures involving mice were conducted in compliance with the Institute of Cancer Research guidelines and approved by the ICR Animal Welfare and Ethical Review Body, adhering to the UK Animals (Scientific Procedures) Act 1986.

Note that full information on the approval of the study protocol must also be provided in the manuscript.

Plants

Seed stocks	No plant species were used.
Novel plant genotypes	No plants species were used.
Authentication	No plants species were used.
